# Therapeutic Potential of Plant-Derived Compounds and Plant Extracts in Rheumatoid Arthritis—Comprehensive Review

**DOI:** 10.3390/antiox13070775

**Published:** 2024-06-27

**Authors:** Mateusz Kciuk, Anjali Garg, Manni Rohilla, Rishabh Chaudhary, Sanchit Dhankhar, Sachin Dhiman, Seema Bansal, Monika Saini, Thakur Gurjeet Singh, Samrat Chauhan, Somdutt Mujwar, Adrianna Gielecińska, Renata Kontek

**Affiliations:** 1 Department of Molecular Biotechnology and Genetics, Faculty of Biology and Environmental Protection, University of Lodz, Banacha St. 12/16, 90-237 Lodz, Poland; 2 Chitkara College of Pharmacy, Chitkara University, Rajpura 140401, Punjab, India; 3 Swami Devi Dyal College of Pharmacy, Golpura Barwala, Panchkula 134118, Haryana, India; 4 Swami Vivekanand College of Pharmacy, Ram Nagar, Banur 140601, Punjab, India; 5 M. M. College of Pharmacy, Maharishi Markandeshwar (Deemed to be University), Mullana, Ambala 133206, Haryana, India; 6 Doctoral School of Exact and Natural Sciences, University of Lodz, Banacha Street 12/16, 90-237 Lodz, Poland

**Keywords:** rheumatoid arthritis, inflammation, natural compounds, oxidative stress

## Abstract

Rheumatoid arthritis (RA) is a persistent autoimmune disorder that is characterized by joint inflammation, discomfort, and impairment. Despite the existence of several therapeutic approaches, their effectiveness is often restricted and may be linked to unfavorable side effects. Consequently, there has been growing interest in investigating naturally derived compounds as plausible therapeutic agents for RA disease. The objective of this review is to summarize the existing preclinical and clinical evidence regarding the efficacy of naturally extracted compounds and plant extracts in the treatment of RA, focusing on their anti-inflammatory, anti-oxidative, and immunomodulatory properties. Some of the problems with using natural chemicals are the uneven quality of commercially available preparations and the poor bioavailability of these compounds. Future investigations should focus on improving the formulations, conducting thorough clinical trials, and exploring different techniques to fully utilize the intrinsic potential of naturally derived chemicals in treating RA.

## 1. Introduction

Rheumatoid arthritis (RA) is a relatively common chronic autoimmune disorder that primarily affects synovial joints, leading to inflammation, pain, swelling, stiffness, and eventually loss of joint function [1]. RA represents a classic example of an autoimmune disease. It is defined by the presence of several forms of autoantibodies, including rheumatoid factor (RF) and anti-citrullinated peptide/protein antibodies (ACPA), along with other abnormalities of the immune system. These markers have strong predictive value and are included in the classification criteria for RA [2,3].

**Figure 1:**
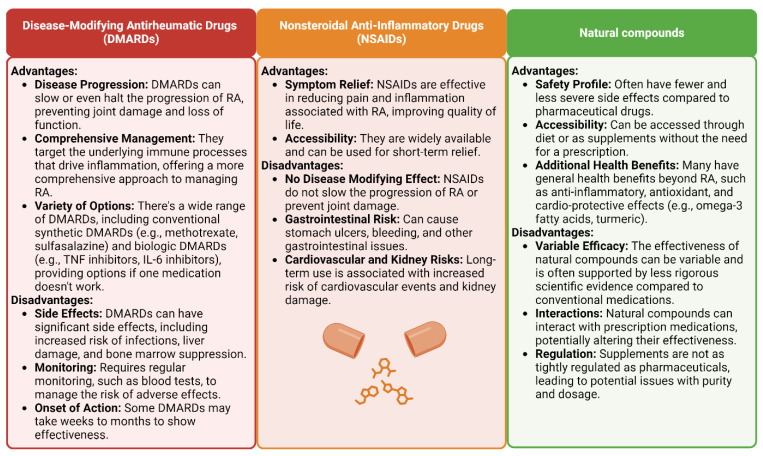
Management of rheumatoid arthritis (RA) using disease-modifying anti-rheumatic drugs (DMARDs), nonsteroidal anti-inflammatory drugs (NSAIDs), and natural compounds. Choosing the right treatment for RA often involves balancing the potential benefits of symptom control against the risk of side effects. DMARDs are central to modifying the disease course and preventing joint damage but come with a significant need for patient monitoring and side effects. NSAIDs are useful for symptom management but do not alter disease progression and carry risks with long-term use. Natural compounds offer a potentially safer profile but may lack the robust evidence supporting their use in disease management. An integrated approach, often combining these treatments under the guidance of healthcare professionals, is typically the most effective strategy for managing RA. Created with BioRender.com, accessed on 3 April 2024.

Approximately one percent of the world’s population lives with RA, and the disease constitutes a significant health burden for the affected individuals and their quality of life. While traditional treatments such as disease-modifying anti-rheumatic drugs (DMARDs) and nonsteroidal anti-inflammatory drugs (NSAIDs) are used to control symptoms, reduce joint inflammation, minimize joint damage, and improve physical function, their use is associated with potential disadvantages and side effects. For example, NSAIDs can cause stomach irritation, ulcers, and bleeding in the gastrointestinal tract. This risk can be higher in older adults or in individuals who take these medications for a prolonged period of time. Long-term use of some NSAIDs has also been associated with an increased risk of heart attack, stroke, and kidney failure. Similarly, conventional DMARDs, such as methotrexate, sulfasalazine, and leflunomide, can cause liver damage, necessitating regular blood tests to monitor liver function. Furthermore, drugs such as methotrexate can cause bone marrow suppression, leading to reduced blood cell counts and increasing the risk of infections and anemia (Figure 1) [4,5,6,7].

Cytokines with pro-inflammatory properties, including tumor necrosis factor-alpha (TNF-α) and interleukin-6 (IL-6), are essential in the onset and spread of inflammatory responses in which immune cells such as T cells, B cells, and macrophages participate. Biological DMARDs (e.g., TNF-α or IL-6 inhibitors) significantly suppress the immune system, raising the risk of serious bacterial, viral, and fungal infections, Furthermore, biological DMARDs are often much more expensive than conventional DMARDs, which can be a significant disadvantage for patients without adequate insurance coverage [8,9].

Lately, there has been a growing interest in studying naturally occurring chemicals as potential treatment agents for RA. Phytochemicals obtained from botanical sources have been utilized for medicinal purposes since ancient times and are deemed to possess a reduced likelihood of side effects in comparison to chemically synthesized pharmaceuticals. In addition, they offer a diverse array of bioactive compounds that have the potential to exhibit anti-inflammatory, immunomodulatory, and analgesic properties [10]. The utilization of naturally extracted compounds has garnered significant interest owing to their capacity to effectively target many pathogenic features associated with RA, as discussed in this review.

This work aims to outline the current information on the possible effectiveness of chemicals obtained from natural sources and their extracts in the treatment of RA, including an analysis of their mechanisms of action and the results of their preclinical investigations and clinical trials. Furthermore, it discusses the difficulties linked with the utilization of natural compounds for RA treatment, encompassing concerns on formulation, bioavailability, and standardization. Furthermore, it provides perspectives on the potential of naturally occurring compounds as alternative or adjuvant therapeutic strategies for managing RA and may contribute to the advancement of innovative therapeutic approaches aimed at enhancing the quality of life and overall prognosis of individuals afflicted by RA.

## 2. Pathophysiology of RA

RA is distinguished by a multifaceted interaction of genetic, environmental, and immunological factors that collectively contribute to its etiology. Genetic predisposition plays a substantial role in the pathogenesis of RA. The heritability of RA has been estimated to be around 60%, with the contribution of human leukocyte antigen (HLA) to heredity estimated to be between 11% and 37%. In addition to the well-known shared epitope (SE) alleles such as HLA-DRB1*01 and DRB1*04, other HLA alleles such as HLA-DRB1*13 and DRB1*15 have been associated with an increased risk of developing RA. A novel categorization system divides SE alleles into four groups: S1, S2, S3P, and S3D. It has been observed that the S2 and S3P groups are predominantly linked to an increased susceptibility to seropositive RA, while the following single nucleotide polymorphisms (SNPs) are the most significant non-HLA gene variants related to RA: phosphatase non-receptor type 22 (PTPN22), interleukin-23 receptor (IL23R), TNF receptor-associated factor 1 (TRAF1), cytotoxic T-lymphocyte-associated protein 4 (CTLA4), interferon regulatory factor 5 (IRF5), signal transducer and activator of transcription 4 (STAT4), chemokine receptor 6 (CCR6), and protein-arginine deiminase type-4 (PADI4). Extensive genome-wide association studies (GWAS) have discovered over 150 specific genomic locations that have a role in the development of RA. HLA genes, together with certain non-HLA genes, can distinguish between individuals with ACPA seropositive RA and those with seronegative RA [2,11,12].

Environmental triggers are thought to initiate or worsen the progression of RA in individuals who are genetically predisposed. These factors may include infections, smoking, and hormonal fluctuations. Various infections, such as those caused by viruses and bacteria, have been linked to the onset of an autoimmune reaction and the consequent induction of RA in individuals who are predisposed to the condition [13,14,15]. The pathogenesis of RA involves a deregulated immune response, whereby self-antigens present in the synovial joint tissues are erroneously identified as foreign, resulting in an atypical immune reaction [16]. The precise cause of this deregulation remains incompletely comprehended, although it is hypothesized to involve a disruption in immune tolerance mechanisms. The activation of immune cells, particularly CD4+ T cells, plays a crucial role in the development of RA. Within the synovial joints, antigen-presenting cells (APCs) present endogenous antigens, which can be recognized by T lymphocytes. Once recognized, pro-inflammatory cytokines such as TNF-α, IL-1, and IL-6 are produced. These cytokines are essential for inflammatory processes, immune cell recruitment and activation, or immune response maintenance in synovial tissue [17,18,19].

**Figure 2:**
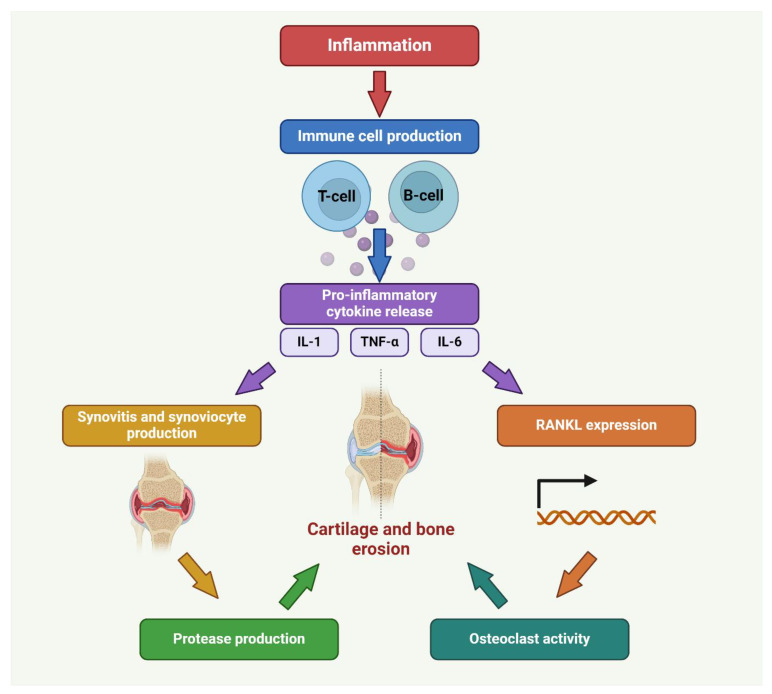
The mechanism underlying the degradation of cartilage and bone as a consequence of inflammation. RA is characterized by erosive damage to the bones. More precisely, the degradation of bones happens as a result of an autoimmune inflammatory response that involves the infiltration of immune cells releasing pro-inflammatory cytokines and primarily targets the synovium, resulting in the deformation of joints. During the acute phase, the synovial membrane releases fluid, experiences increased blood flow, and swells as a result of the increased protease and osteoclast activity (due to increased receptor activator for nuclear factor κB ligand (RANKL) expression in response to inflammatory cytokines). RANKL is highly involved in osteoclast production, which degrades the bone structure. During the chronic stage, the sliding surfaces undergo hypertrophy and develop fluffy protrusions known as pannus. These protrusions extend into the joint cavity or penetrate the cartilage and subchondral bone. This presents as joint stiffness, discomfort, soreness, and swelling. During the advanced stage, there is a degeneration of the muscles around the joints, resulting in the fingers deviating towards the ulnar side. Additionally, there is a partial dislocation of the metacarpophalangeal joint, leading to the characteristic appearance of “swan neck” and “button patterns” [22]. Created with BioRender.com, accessed on 3 April 2024.

The pathogenesis of RA involves the activation of B cells, which subsequently results in the production of autoantibodies. Activated B cells undergo a process of differentiation, resulting in the generation of plasma cells. Plasma cells are integral in the production of autoantibodies, including RF and anti-CCPs. Autoantibodies bind to self-antigens within the synovium, resulting in the infliction of damage to tissues and the perpetuation of the inflammatory response. The development of pannus can occur as a result of prolonged immune activation, leading to chronic inflammation of the synovial tissue [20,21] shown in Figure 2. The synovial membrane, a thin tissue layer that surrounds the joints, experiences hypertrophy and thickening, resulting in the development of a pannus.

**Figure 3:**
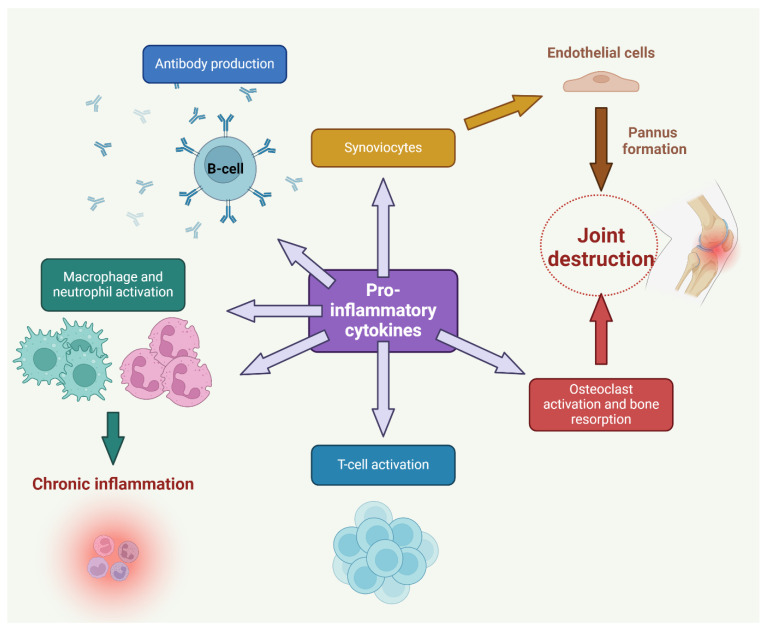
Pannus formation (an aggressive growth of synovial tissue) plays a vital role in the pathophysiology of rheumatoid arthritis (RA). This graphic emphasizes several key components involved in the formation of the pannus and the subsequent deterioration of joints, including pro-inflammatory cytokines, macrophages, antibody synthesis, neutrophils, synoviocytes, and osteoclasts. Created with BioRender.com, accessed on 3 April 2024.

The pannus is a tissue characterized by the infiltration of synovial fibroblasts, inflammatory cells, and neovascularization. It demonstrates a highly assertive demeanor as it infiltrates and erodes both the cartilage and subchondral bone, leading to the deterioration and deformation of the joint [23]. RA affects more than just the joints; it also affects the skin, heart, lungs, kidneys, eyes, and blood vessels, among other organs and tissues. The systemic complications associated with RA are a significant contributor to the overall disease burden and pose a heightened risk of morbidity and mortality in affected patients [7]. Comprehending the intricate pathophysiological mechanisms underlying RA is of paramount importance in the development of precise therapeutic strategies and interventions. It is plausible that the modulation of disease activity and prevention of joint damage in individuals with RA can be achieved by targeting particular components of the immune response and inflammatory pathways, as shown in Figure 3.

## 3. Natural Compounds as Anti-RA Agents—Overview

Throughout human history, various endeavors have been made to alleviate the symptoms of rheumatism by harnessing the therapeutic properties of naturally existing substances. The objective of these investigations was to evaluate the efficacy of naturally occurring compounds in mitigating bone deterioration in individuals diagnosed with RA, utilizing diverse treatment modalities. The capacity of naturally derived compounds to impede the activation of inflammatory mediators, including cytokines, enzymes, and transcription factors, is responsible for their anti-inflammatory properties [24]. Through modulation of T cell differentiation and activity, or the generation of regulatory T cells (Tregs), inhibition of B cell activity (that produces autoantibodies and immune complexes linked to RA), natural compounds alleviate the inflammatory response. Furthermore, their impact on inflammatory signaling pathways such as nuclear factor kappa B (NF-κB), mitogen-activated protein kinases (MAPKs), and Janus kinase/signal transducers and activators of transcription (JAK/STAT) pathways can impede the progression of RA disease [25].

The pathogenesis of RA is significantly influenced by oxidative stress, which contributes to inflammation and tissue damage. Compounds extracted from natural sources have been found to possess significant anti-oxidant properties, effectively scavenging free radicals and mitigating oxidative stress. The elevated activity of endogenous anti-oxidant enzymes, such as superoxide dismutase (SOD) and glutathione peroxidase (GPX), can result in the safeguarding of cells against oxidative damage [26,27,28,29].

## 4. Plant-Derived Compounds Investigated in Clinical Trials

### 4.1. Curcumin

Turmeric (*Curcuma longa*) is a commonly used spice in Asian cuisine and traditional medicine that contains curcumin as its major bioactive compound. Curcumin has several medical uses; it is the bioactive component that gives turmeric its distinctive bright yellow color and has anti-inflammatory, anti-oxidant, and immunomodulatory actions. Curcumin is a polyphenolic compound characterized by a structurally intricate chemical composition. It is classified as a constituent of the natural compound category known as curcuminoids, which includes demethoxycurcumin and bisdemethoxycurcumin. The aforementioned constituents are accountable for the distinctive chemical characteristics of turmeric and its biological properties, as they may exert synergic effects [30,31,32].

The beneficial impact of curcumin on RA is believed to be mediated through its anti-inflammatory, anti-oxidant, and immunomodulatory properties. While the exact molecular mechanisms are not fully understood, several key pathways and processes have been proposed to contribute to curcumin’s effects on RA. Curcumin has been shown to inhibit the production of various inflammatory mediators involved in the pathogenesis of RA, including cytokines such as TNF-α, IL-1β, and IL-6. By blocking the production or activity of these pro-inflammatory cytokines, curcumin can help reduce inflammation and attenuate the inflammatory response in RA [33,34,35,36]. NF-κB is a key transcription factor that regulates the expression of genes involved in inflammation, immune response, and cell survival. Curcumin has been shown to inhibit NF-κB activation by blocking the phosphorylation and degradation of its inhibitory protein, NF-kappa-B inhibitor alpha (IκBα), thereby preventing the translocation of NF-κB to the nucleus and subsequent transcription of pro-inflammatory genes [37,38]. Curcumin can also modulate various cellular signaling pathways implicated in RA pathogenesis, including 5′-AMP-activated protein kinase catalytic subunit alpha-1 (AMPK)-MAPK, protein kinase C (PKC) [39], phosphatidylinositol 4,5-bisphosphate 3-kinase-AKT serine/threonine kinase (PI3K-AKT) [33], and JAK-STAT signaling [40]. By interfering with these signaling pathways, curcumin can regulate immune cell activation, cytokine production, and inflammatory responses in RA. It was also shown to induce apoptosis in synovial cells, including fibroblast-like synoviocytes (FLS), which play a key role in the hyperplasia and invasive behavior of the synovium in RA. By promoting apoptosis of synovial cells, curcumin may help reduce synovial inflammation, pannus formation, and joint destruction in RA [41,42]. Oxidative stress and reactive oxygen species (ROS) play a role in the pathogenesis of RA by promoting inflammation and tissue damage. Curcumin exhibits potent anti-oxidant activity and can scavenge free radicals, reduce oxidative stress, and protect cells from oxidative damage in RA [43,44].

**Figure 4:**
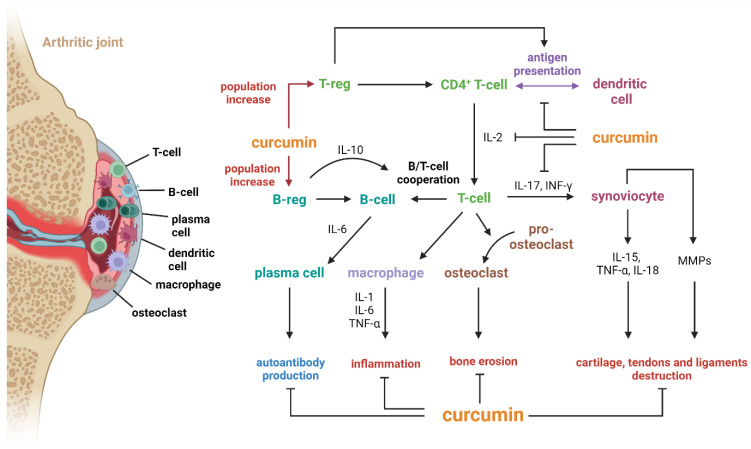
The immunomodulatory effects of curcumin on dysregulated immune cells in rheumatoid arthritis (RA). Curcumin suppresses the synthesis of certain crucial pro-inflammatory cytokines, including tumor necrosis factor-alpha (TNF-α) and interleukins (IL-1β and IL-6), by activated macrophages. Curcumin also inhibits the activation and production of cytokines by autoreactive T cells, which subsequently leads to the reduced activation of pathogenic synoviocytes and osteoclasts. Curcumin restores the balance between TH17 and regulatory T (Treg) cells, leading to a rise in the number of Treg cells and the subsequent production of anti-inflammatory cytokines such as IL-10. Curcumin inhibits the generation of autoantibodies by auto-reactive B cells and increases the population of B regulatory (Breg) cells, which decreases T cell immunological responses. Ultimately, curcumin’s immunomodulatory effects result in the prevention of joint and bone damage in RA. Based on [45]. Created with BioRender.com, accessed on 3 April 2024.

Additionally, curcumin has immunomodulatory effects and can regulate immune cell function, including T cells, B cells, macrophages, and dendritic cells. By modulating immune responses, curcumin may help restore immune homeostasis and suppress aberrant immune activation and inflammation in RA. The possible immunomodulatory mechanisms of curcumin activity in RA obtained from pre-clinical and clinical studies were recently summarized by Haftcheshmeh Mohammadian et al. [45] and are shown in Figure 4.

Despite showing significant therapeutic potential, curcumin’s practical application has been restricted by its inherent low bioavailability and fast metabolism. To enhance the water solubility, stability, and possible uses of curcumin/curcuminoids, several approaches have been suggested and studied. These include the use of nanoparticles, liposomes, solid dispersions, solid lipid nanoparticles, microemulsions, and the formation of complexes with phospholipids and cyclodextrins [46,47].

When examining the impact of natural chemicals on rheumatoid arthritis (RA), it is essential to differentiate between various types of research studies: in vitro assays, in vivo assays, and clinical trials. These differences impact how the results are understood and how they can be applied to human health. In vitro assays are scientific investigations performed in a controlled setting separate from a living organism, usually in petri dishes or test tubes, utilizing cells or biological materials. In vitro investigations enable researchers to investigate the precise cellular-level mechanisms of action of natural substances. These tests are frequently employed for primary screening to detect the possible therapeutic benefits and toxicity of substances. The controlled environment minimizes fluctuations, enabling accurate measurements, but may not completely duplicate the intricacy of living organisms [48,49,50].

Animal models commonly used to study human rheumatoid arthritis include collagen-induced arthritis (CIA), adjuvant-induced arthritis (AIA), human TNF transgenic mice, streptococcal cell wall-induced arthritis, proteoglycan-induced arthritis, and serum transfer-induced models. These models are utilized to examine the specific components and stages of the inflammatory process and assess the effectiveness of newly developed therapies in preclinical settings. The AIA and CIA models are extensively used in research on RA. These models accurately mimic the clinical features of RA, including joint inflammation, swelling, hyperplasia of synovial tissue, and loss of cartilage and bone [50,51,52,53,54].

In contrast, in clinical trials evaluating the response to anti-RA drugs, several parameters are commonly used to assess the efficacy of the treatment. These include:

(1) Disease Activity Scores: Disease activity scores such as the Disease Activity Score in 28 Joints (DAS28) or Clinical Disease Activity Index (CDAI) are commonly used to assess the overall disease activity in RA patients. These scores incorporate measures of joint tenderness and swelling, acute-phase reactants (e.g., C-reactive protein (CRP) or Erythrocyte Sedimentation Rate (ESR)), and patient-reported outcomes such as pain and global assessment of disease activity.

(2) Joint Assessments: Clinical trials often involve assessments of individual joints to evaluate changes in swelling, tenderness, and range of motion. The number of tender and swollen joints may be recorded and analyzed to determine the treatment response. The tender joint count (TJC) is a clinical assessment used to evaluate the number of joints that are tender to palpation in a patient with RA. The TJC specifically focuses on the tenderness of joints, typically involving the examination of a predefined set of joints, such as those in the hands, wrists, elbows, shoulders, knees, and ankles. The swollen joint count (SJC) also belongs to the category of joint assessments, just like the TJC. The SJC is used to evaluate the number of joints that are swollen due to inflammation.

(3) Acute-Phase Reactants: Levels of acute-phase reactants such as CRP and ESR are frequently measured in clinical trials. Reductions in these markers indicate a decrease in inflammation and disease activity. In contrast, RF is an autoantibody that targets the Fc portion of immunoglobulin G antibodies, leading to immune complex formation and inflammation in the joints and other tissues.

(4) Patient-Reported Outcomes: Patient-reported outcomes (PROs) provide valuable insights into the impact of treatment on patients’ quality of life and functional status. PROs may include assessments of pain, fatigue, physical function, and health-related quality of life. The Visual Analog Scale (VAS) is a PRO measure commonly used in clinical trials and clinical practice to assess various aspects of health, including pain intensity, disease activity, and other subjective experiences. It is typically presented as a horizontal or vertical line with endpoints representing extreme states (e.g., “no pain” to “worst pain imaginable” for pain intensity). The Western Ontario and McMaster Universities Osteoarthritis Index (WOMAC) is a widely used questionnaire designed to assess the severity of osteoarthritis (OA) symptoms and physical function in individuals with hip or knee osteoarthritis. The Numeric Pain Rating Scale (NPRS) is a commonly used tool for assessing pain intensity, where patients rate their pain on a scale from 0 to 10, with 0 indicating no pain and 10 indicating the worst possible pain. It allows patients to provide a subjective assessment of their pain levels, making it a valuable measure in clinical practice and research for monitoring pain severity, evaluating treatment effectiveness, and assessing changes in pain over time.

(5) Radiographic Assessments: X-rays or other imaging modalities may be used to assess changes in joint damage and the progression of structural joint damage over time. Reductions in joint erosions and improvements in radiographic scores indicate a beneficial effect on disease progression.

(6) Composite Measures: Composite measures such as the American College of Rheumatology (ACR) response criteria or the European League Against Rheumatism (EULAR) response criteria combine multiple parameters to evaluate the overall treatment response. These criteria categorize patients into different response levels (e.g., good, moderate, or no response) based on predefined thresholds.

(7) Functional Assessments: Functional assessments, including measures of physical function and disability (e.g., Health Assessment Questionnaire Disability Index, HAQ-DI), assess the impact of treatment on patients’ ability to perform daily activities and participate in social roles.

(8) Safety Assessments: In addition to efficacy outcomes, clinical trials also evaluate the safety and tolerability of anti-RA drugs. Adverse events, laboratory abnormalities, and other safety parameters are monitored throughout the trial.

By incorporating these parameters into clinical trial designs, researchers can comprehensively assess the efficacy, safety, and impact of anti-RA drugs on disease activity, symptoms, functional status, and structural damage in RA patients [55,56,57,58,59,60,61,62,63,64,65,66].

In a randomized pilot study, a total of forty-five patients diagnosed with RA were randomly assigned to one of three groups. The patients in each group were given either curcumin (500 mg in the form of BCM-95W, a patented and registered formulation of curcumin that has been developed to have improved bioavailability), diclofenac sodium (50 mg), or a combination of both. The main objective of the study was to measure the decrease in DAS28. The secondary outcomes encompassed the use of ACR standards to assess the decrease in soreness and swelling of joints. All three treatment groups exhibited statistically significant alterations in DAS measurements. Remarkably, the group treated with curcumin had the greatest percentage of improvement in both total DAS and ACR scores (ACR 20, 50, and 70), and these scores were considerably superior to those of the patients in the diclofenac sodium group. Significantly, the administration of curcumin was determined to be safe and did not exhibit any harmful effects [67].

A clinical trial was performed to assess the relative effectiveness of two different dosages of curcumin compared to a placebo in patients with active RA. The study followed a randomized, double-blind, placebo-controlled design, with three groups receiving different treatments simultaneously. A total of twelve patients were assigned to each group and administered either a placebo, 250 mg, or 500 mg of the curcumin product twice daily for 90 days. The patients’ reactions were evaluated using the ACR response, VAS, CRP, DAS28, ESR, and RF values. Patients with RA who were administered the curcumin product at both low and high doses experienced statistically significant improvements in their clinical symptoms by the end of the research. The trial product led to substantial changes in ESR, CPR, and RF values compared to baseline and placebo, confirming the observations. The findings suggest that this new form of curcumin functions as a pain-relieving and anti-inflammatory substance for treating RA. The effective dosage is as low as 250 mg, taken twice a day. Both administrations of the experimental treatment were well received by the participants and did not result in any adverse reactions [68].

Another clinical trial assessed the effect of curcumin nanomicelles administered to RA patients three times a day for 12 weeks. DAS28 and ESR responses were evaluated following the 12 weeks. There were no significant differences in the DAS-28, TJC, and SJC at the beginning and end of the research between the curcumin nanomicelle and the control groups. However, the shift was more significant in the experimental group compared to the placebo group. There were no notable differences in terms of ESR between the two groups of RA patients [69].

Another randomized, double-blind, placebo-controlled clinical trial was conducted in RA women to examine the impact of curcumin supplementation (500 mg once a day or placebo for 8 weeks) on metabolic parameters, such as lipid profile and glycemic indices, as well as inflammatory variables, visfatin levels, and obesity values. Supplementing with curcumin resulted in significant improvements in various health markers, including insulin resistance, inflammation, levels of CRP and triglycerides, body mass index, and diameter of the waist, in patients compared to those who received placebo at the end of the study. The placebo group experienced a significant increase in the Homeostatic Model Assessment of Insulin Resistance (HOMA-IR) and lipid levels. There were no significant changes observed in fasting blood sugar, insulin, other lipid profiles, or visfatin levels in any of the groups. The findings of this study provide evidence for the beneficial effects of consuming curcumin as a component of a comprehensive strategy to regulate metabolic variables, inflammation, and obesity in women with RA [70].

Another study investigated the effect of exercise and curcumin supplementation on RA patients. A total of ninety patients were assigned randomly to two groups. One group received treatment consisting of both strengthening activities and curcumin, while curcumin was administered purely to another group. Both groups were administered curcumin orally at a dosage of 180 mg per day. The first group received a treatment regimen consisting of three sessions per week, with each session lasting 45 min. Additionally, serological results and X-rays of the joints were conducted for evaluation purposes. The assessment of pain, morning stiffness, and functional activities was conducted using the WOMAC and NPRS scales at the beginning, 12th week, and 24th week. The first group exhibited a statistically significant decrease in the quantitative values of RF, ESR, CRP, WOMAC pain, and stiffness [71].

Although preclinical studies and some small-scale clinical trials have shown positive effects of curcumin in RA, the evidence is still limited and inconclusive [72,73,74]. Larger, well-designed clinical trials are needed to confirm these findings and determine the efficacy and safety of curcumin in RA management. The optimal dosing of curcumin for therapeutic effects in RA has not been established. Further, clinical trials are needed to evaluate different doses and formulations of curcumin to determine the most effective and safe regimen for RA patients. Curcumin has poor bioavailability, meaning that it is poorly absorbed and rapidly metabolized in the body, which may limit its effectiveness [74]. Clinical trials investigating novel formulations or delivery methods of curcumin, such as nanoformulations or combination therapies, are warranted to enhance its bioavailability and therapeutic efficacy in RA [75,76,77,78]. While curcumin is generally considered safe and well-tolerated, its long-term safety profile in RA patients has not been fully established. Further clinical trials are needed to assess the safety of prolonged curcumin use, including potential adverse effects and drug interactions, particularly in combination with conventional RA treatments [79]. There are currently no clinical trials evaluating the effects of curcumin treatment in RA patients (source: https://clinicaltrials.gov/search?cond=Rheumatoid%20Arthritis&intr=Curcumin accessed on 3 April 2024).

Nevertheless, turmeric and curcumin do not cause mutations or damage to genetic material. The oral administration of turmeric and curcumin in mice did not result in any adverse effects on the reproductive system. Research conducted on humans found no evidence of harmful effects, indicating that curcumin is safe when taken orally at a dosage of 6 g/day for 4–7 weeks. However, curcumin may elevate the amount of oxalate in the urine, which can raise the likelihood of kidney stone formation in persons who are genetically predisposed to kidney stone formation. Caution should be taken while using high-dose curcumin in patients with subclinical iron insufficiency, chronic anemia, and consumptive anemia produced by heavy burdens of malignant tumors [80]. Lukefahr et al. [81] have also documented a case of drug-induced autoimmune hepatitis caused by the consumption of curcumin supplements. While there is less documentation on liver damage specifically caused by curcumin, the available findings highlight the significance of curcumin supplementation as a catalyst for drug-induced liver disease, as extensively reviewed by Liu et al. [80].

Nevertheless, it has been determined that oral bioavailable formulations of curcumin are deemed safe for human consumption at a dosage of 500 mg twice a day for 30 days. However, there are a limited number of trials conducted, and further research is required, particularly focusing on nanoformulations [82,83,84]. More research is needed to elucidate the mechanisms underlying the anti-inflammatory and immunomodulatory effects of curcumin in RA. Clinical trials incorporating biomarker analysis and imaging studies can help provide insights into the molecular pathways and biological processes involved in curcumin’s therapeutic effects in RA.

### 4.2. Boswellic Acids

**Figure 5:**
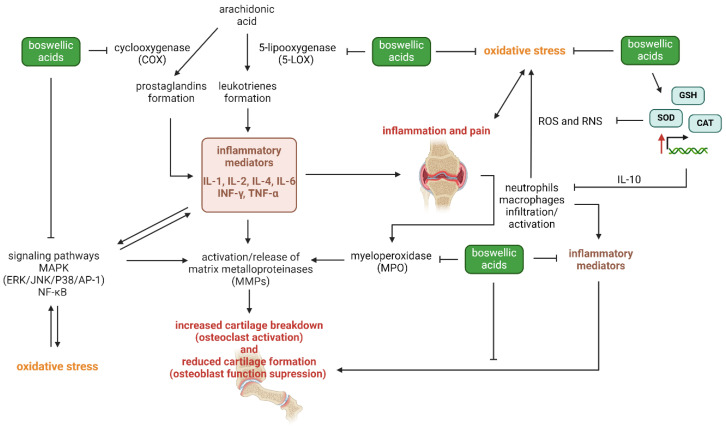
The schematic representation of boswellic acid activity in RA treatment. A detailed description was provided in the main text of the manuscript. AP-1—adaptor protein 1 ERK—extracellular signal-regulated kinase; MAPK—mitogen-activated protein kinase; P38—mitogen-activated protein kinase p38; ROS—reactive oxygen species; RNS—reactive nitrogen species. Created with BioRender.com, accessed on 3 April 2024.

Boswellic acids encompass a collection of biologically active compounds that originate from the resin of *Boswellia serrata*, a botanical species widely recognized as Indian frankincense. Numerous studies have investigated the medicinal properties of Boswellic acids, including β-boswellic acid, acetyl-β-boswellic acid, 11-keto-β-boswellic acid, and their derivatives. Boswellic acids hinder the production of leukotrienes in neutrophilic granulocytes by impeding the activity of 5-lipoxygenase and cyclooxygenases (COX) [85]. In addition, boswellic acids have been reported to hinder elastase activity in leukocytes [86,87] and impact the cellular defense system through their interaction with the generation and release of cytokines through the suppression of the NF-κB pathway and a decrease in proinflammatory cytokines such as TNF-α, IL-1, IL-2, IL-4, IL-6, and IFN-γ [88]. Furthermore, boswellic acids decreased myeloperoxidase activity (MPO) activity and nitric oxide production (NO) by the immune cells while enhancing catalase (CAT) and SOD activity and glutathione (GSH) production in joints of rats immunized with collagen type II, decreasing inflammation mediated by IL-1β, IL-6, TNF-α, interferon-γ (INF-γ), and prostaglandin E2 (PGE2) and protecting cells through increased levels of IL-10 [89]. MPO is an enzyme involved in the generation of ROS, contributing to oxidative stress and tissue damage in inflammatory conditions [90]. By decreasing MPO, boswellic acids reduce oxidative stress and lipid peroxidation, protecting joint tissues from damage. NO is a signaling molecule that plays a role in inflammation. Although it has various physiological functions, excessive NO production can contribute to inflammation and joint damage in RA [91]. Boswellic acids’ ability to reduce NO production helps mitigate inflammation and joint destruction. CAT and SOD are critical anti-oxidant enzymes that neutralize ROS, while GSH is a major anti-oxidant that protects cells from oxidative damage [92]. By enhancing the activity of these enzymes and increasing GSH production, boswellic acids strengthen the anti-oxidant defense system, reducing oxidative stress and inflammation in the joints. Pro-inflammatory cytokines (IL-1β, IL-6, TNF-α, and IFN-γ) and PGE2 are key mediators of inflammation and bone erosion in RA. They stimulate osteoclastogenesis (the formation of bone-resorbing cells) and inhibit osteoblast function (the cells responsible for bone formation), leading to bone erosion. PGE2 also contributes to inflammation and bone remodeling processes. Conversely, IL-10 is an anti-inflammatory cytokine that can counteract the effects of pro-inflammatory cytokines and promote the resolution of inflammation. By shifting the balance towards anti-inflammatory responses, boswellic acids help reduce inflammation and protect against bone erosion and joint damage [88,89]. The mechanism of action of boswellic acids in RA is shown in Figure 5.

Although boswellic acids have favorable properties in treating chronic diseases by targeting many pathways and their beneficial effects on the complement system [86,93,94] pharmacokinetic qualities are a significant concern, resulting in suboptimal pharmacological efficacy [95]. This is due to the low oral bioavailability, partially due to the high lipophilicity, rapid metabolism, and poor intestinal absorption [95]. The subsequent investigations focused on exploring several ways to create boswellic acid nanoparticles and evaluating their therapeutic benefits. Various advanced drug delivery systems, including polymeric micelles, metallic nanoparticles (ZnO and Ag), polymeric nanoparticles (chitosan and lactic-co-glycolic acid), nano metal-organic frameworks (zeolitic imidazolate framework-8), phytosomes, liposomes, and nanogels, are utilized to create boswellic acid nanoparticles. These systems are employed to enhance the oral bioavailability and pharmacokinetic properties of natural compounds [96].

Nevertheless, boswellic acid use is generally considered safe. The first studies appeared in 1996. No deaths occurred in rats or mice when given boswellic acids orally or intraperitoneally at doses up to 2 g/kg. Administering BAs orally to rats and monkeys at three different dosages (125, 250, and 500 mg/kg) daily for six months did not result in any significant alterations in overall behavior or in clinical, hematological, biochemical, or pathological measurements [97]. No mortality or symptoms of toxicity were observed in Wistar rats exposed to a maximum dose of 2000 mg/kg of *Boswellia serrata* gum resin extract, as indicated by acute oral and acute cutaneous toxicity experiments. The extract did not elicit any irritation on the skin or eyes of New Zealand white rabbits. In a 28-day oral toxicity trial involving repeated doses, Wistar rats treated with the extract did not exhibit any evidence of toxicity, as no changes in their body weights, organ weights, or hematological and clinical chemistry parameters were noted. The estimated no observed negative effect level (NOEL) for *Boswellia serrata* gum resin extract was 1000 mg/kg/day in male and female rats. The extract did not show any mutagenic or clastogenic effects, as determined by the bacterial reverse mutation test and micronucleus assay in mouse bone marrow erythrocytes [98]. In a randomized, double-blind, placebo-controlled trial, the participants were instructed to take two tablets of 169.33 mg of *Boswellia serrata* extract daily, with each tablet carrying an average of 87.3 mg of total β-boswellic acids for 120 days. There were no adverse effects associated with the use of boswellic acids [99].

In preclinical studies, combined use of 10% acetyl keto boswellic acid (AKBA) with methotrexate (MTX) effectively inhibited arthritis and prevented the hepatotoxicity caused by MTX in arthritic mice [100]. Choudhary et al. [101] later investigated the anti-arthritic properties of BA-25 (3-α-o-acetoxy-4β-amino-11-oxo-24-norurs-12-ene), an amino analog of β-boswellic acid combined with MTX and MTX alone. The suppression of NO, ROS, and pro-inflammatory cytokines such as TNF-α and IL-6 was shown in lipopolysaccharide (LPS)-stimulated RAW-264.7 cells when the combined treatment was compared to MTX alone. The combination’s ability to suppress cytokines was further confirmed in vivo using balb/c mice, where it successfully reversed the increase in pro-inflammatory cytokines induced by LPS. The toxicological effects of the combination were examined in mice for 28 days. The results indicated that there were no significant alterations in the hematological and serum biochemical parameters between the combination group and the control group. Attenuation of liver toxicity was observed when the agents were used together. Additionally, BA-25 downregulated the expression of the apoptotic protein BAX while raising the expression of the anti-apoptotic protein BCL2. Additionally, pharmacokinetic investigations were carried out on BA-25 in Balb/c mice, revealing its fast absorption. The authors also examined the anti-arthritic impact of combining MTX + BA-25 compared to using MTX alone in a CIA model of DBA/1 mouse disease. The results showed that treating animals with the combination significantly reduced paw inflammation as well as levels of IL-6 and IL-1β. In addition, suppression of the NF-κB pathway in the ankle-joint tissue of mice treated with the combination therapy was found. The findings suggested that combination of MTX with BA-25 had a greater ability to reduce inflammation and alleviate arthritis, as demonstrated by both laboratory experiments and research conducted on living organisms [101].

The first reports on the clinical use of boswellic acids in the treatment of RA date back to 1996, when the use of H15, a special extract of the gum resin of *Boswellia serrata,* was shown to reduce joint swelling, pain, ESR, and stiffness in RA patients [102]. Despite that, the following investigation of H15 extract in RA patients’ treatment has not demonstrated clinical efficiency [103]. In a randomized, double-blind, placebo-controlled trial, it was found that β-boswellic acid (174.6 mg/in two tablets/day/120 days) treatment in osteoarthritic patients enhanced their physical function by alleviating pain and lowering stiffness in comparison to the placebo group. The radiographic evaluations demonstrated an improved knee joint gap and decreased osteophytes, demonstrating the effectiveness of the treatment. Boswellic acids also markedly decreased the serum concentrations of CRP and did not result in adverse effects [99].

Although preclinical studies have provided evidence for the potential use of boswellic acids in RA treatment, there is a lack of clinical investigations. There is only one clinical study planned to investigate the effectiveness of some natural JAK-STAT inhibitor including boswellic acids, as supplemental therapy for treating RA and reducing the adverse effects of standard MTX treatment (https://clinicaltrials.gov/; NCT05788705; accessed on 3 April 2024).

### 4.3. Resveratrol

Resveratrol is an endogenous polyphenolic compound that is ubiquitously present in diverse botanical sources, including grapes, berries, and peanuts. The compound has attracted attention due to its anti-oxidative and anti-inflammatory properties, as well as its purported ability to enhance overall well-being. Resveratrol is categorized as a stilbene compound and exhibits two chemical configurations: trans-resveratrol and cis-resveratrol. The trans configuration is considered to be the biologically active conformation and is commonly observed in botanical organisms [104]. Several preclinical studies have investigated the anti-inflammatory, anti-oxidant, and immunomodulatory properties of resveratrol and its potential role in the management of RA. Resveratrol exhibits potent anti-inflammatory properties by inhibiting the production and activity of pro-inflammatory mediators, such as cytokines (e.g., TNF-α, IL-1β, IL-6), chemokines, and inflammatory enzymes (e.g., COX-2) [105]. By reducing inflammation, resveratrol may help alleviate joint pain, swelling, and stiffness in RA. While the direct scavenging activity of this substance is relatively low, its anti-oxidant characteristics in living organisms are more likely attributed to its function as a gene regulator. The regulation of the genes by resveratrol is mostly facilitated by either sirtuin 1 (SIRT1) or nuclear factor erythroid 2-related factor 2 (NRF2). SIRT1 is essential for resveratrol to activate AMPK and enhance mitochondrial function both in vitro and in living organisms [106].

Oxidative stress contributes to joint damage, synovial inflammation, and cartilage degradation in RA. By neutralizing free radicals and enhancing anti-oxidant defenses, resveratrol may protect joint tissues from oxidative damage and slow disease progression in RA. Resveratrol modulates immune responses by regulating the activity of immune cells, including T cells, B cells, macrophages, and dendritic cells. It can suppress excessive immune activation and inflammatory cytokine production while promoting regulatory T-cell function and anti-inflammatory cytokine secretion. By restoring immune homeostasis, resveratrol may help attenuate autoimmune responses and reduce inflammation in RA. Resveratrol has been shown to down-regulate matrix metalloproteinases (MMPs) (through the mechanism mediated by SIRT1), enzymes involved in the degradation of extracellular matrix components (including collagen and proteoglycans). MMPs contribute to cartilage destruction and joint damage in RA. By inhibiting MMPs, resveratrol may help preserve joint integrity and prevent irreversible damage. Furthermore, SIRT1 exerts an anti-inflammatory function by suppressing the NF-κB signaling pathway and the synthesis of monocyte-derived inflammatory factors, including TNF-α, IL-1B, and IL-6. Some studies suggest that resveratrol may have protective effects on bone health by promoting osteoblast differentiation and inhibiting osteoclast formation and activity. These effects may help mitigate bone erosion and osteoporosis, common complications of RA, as recently reviewed by other authors [22,107].

Resveratrol has recently been identified as a promising therapeutic for suppressing inflammation in CIA in mouse models. Resveratrol was observed to reduce the levels of TNF-α, IL-1β, IL-6, and monocyte chemoattractant protein 1 (MCP-1) in the blood and joint tissue, as well as the RANKL. The expression of NF-κB was reduced in mice that were fed resveratrol. Therefore, it was determined that adding resveratrol to the diet may help reduce inflammation and bone damage in mice with CIA [108,109]. Furthermore, resveratrol was able to trigger apoptosis in FLS obtained from patients with RA by activating the caspase-8-dependent pathway [110,111].

A total of 100 patients diagnosed with RA (68 female, 32 male) were divided into two groups, each consisting of 50 patients. The first group received a daily capsule containing 1 g of resveratrol in addition to the standard treatment for 3 months. The second group, serving as the control group, received only regular medications: leflunomide, hydroxychloroquine, sulfasalazine, methotrexate, prednisolone, and various nonsteroidal anti-inflammatory drugs. The clinical and biochemical indicators of RA were evaluated in both groups. The study revealed a substantial decrease in the clinical markers, including DAS28 in the group co-treated with resveratrol. In addition, the levels of CRP, ESR, undercarboxylated osteocalcin, MMP-3, TNF-α, and IL-6 were considerably reduced in patients who had resveratrol treatment [112].

While the preclinical evidence supporting the therapeutic potential of resveratrol in RA is promising, further research, including well-designed clinical trials, is needed to fully elucidate its efficacy, safety, optimal dosage, and long-term effects in RA patients [113]. Additionally, considering the relatively low bioavailability of resveratrol, strategies to enhance its absorption and delivery may be necessary to maximize its therapeutic benefits in RA [114]. Both in vivo investigations and clinical trials suggest that high-dosage formulations of resveratrol may have the potential for drug-drug interactions. Resveratrol may interact with different cytochrome P450 (CYP) enzymes, particularly when consumed in high amounts. In addition to systemic CYP inhibition, it is also important to take into account intestinal interactions. They can decrease the first-pass metabolism, leading to increased levels of co-administrated CYP substrates. As a result, those who consume large quantities of this dietary supplement in conjunction with other pharmaceuticals may face the possibility of encountering significant drug-drug interactions with clinical implications [115,116]. Depending on the source and formulation, resveratrol supplements can also be expensive, especially when used at higher doses. Cost may be a barrier to access for some patients, particularly those with limited financial resources or inadequate insurance coverage. Additionally, as a dietary supplement, resveratrol is not subject to the same rigorous regulatory oversight as pharmaceutical drugs. This may lead to variability in product quality, purity, and potency, raising concerns about safety and efficacy.

### 4.4. Quercetin

Quercetin is a flavonoid compound present in a variety of fruits, vegetables, and plants. Quercetin is recognized for its robust anti-oxidative and anti-inflammatory characteristics, which are responsible for its pro-health properties, and exhibits a ubiquitous presence in the natural world in various food sources, including but not limited to apples, onions, berries, citrus fruits, leafy greens, and tea. The quercetin content in various food sources exhibits variability. The health benefits of quercetin are attributed to its anti-oxidative and anti-inflammatory properties, which safeguard cells and tissues against oxidative harm. Quercetin has been the subject of research due to its potential impact on cardiovascular health, immune function, and cancer prevention [117,118,119]. The pre-clinical investigations revealed that quercitin can alleviate the RA symptoms in various mouse models of the disease, including AIA, CIA, oil-induced arthritis, and proteoglycan-induced arthritis, as reviewed by Guan et al. [120].

Out of the many human intervention studies that have been published, there have been very few reports of negative effects from taking quercetin supplements. And even in those cases, the effects were moderate. Currently, scientific data to assess the safety of using high dosages of quercetin supplements (≥1000 mg) for an extended period of time (>12 weeks) is scarce. Animal investigations into the oral use of quercetin have discovered significant safety concerns. These include the possibility of quercetin to increasing nephrotoxicity in individuals with kidney diseases, as well as the potential for promoting tumor growth, particularly in estrogen-dependent cancers. Moreover, both animal and human investigations involving the administration of quercetin as a supplement for a single time or a short time have shown that quercetin can interact with certain medicines, resulting in changes in the way these drugs are absorbed and distributed in the body. This topic was previously reviewed in excellent works by Andres et al. [121], Harwood et al. [122], and Mirza et al. [123].

Despite its various pharmacological advantages, quercetin’s limited utilization as a therapeutic molecule in clinical settings is due to its poor water solubility, considerable first-pass metabolism, and resulting low bioavailability [123]. Recent research indicates that quercitin nanoformulations can significantly address bioavailability concerns in RA. Various nanoformulations, including liposomes, nanogels, micelles, solid lipid nanoparticles, polymer nanoparticles, gold nanoparticles, and cyclodextrin complexes, have demonstrated enhanced stability, cellular bioavailability, and therapeutic efficiency of hydrophobic drugs. These nanoformulations ensure higher encapsulation efficiency, sustained release, prolonged circulation time, and improved accumulation at the target sites, thereby enhancing the therapeutic potential of curcumin in RA patients. Additionally, combining quercitin, with other therapeutic agents in a single nanocarrier has shown the potential to improve treatment outcomes. Thus, quercitin nanoformulations offer a comprehensive strategy to overcome its inherent limitations, ensuring better absorption, distribution, metabolism, excretion, and toxicity profile, ultimately leading to improved clinical application in RA management [120,123,124,125,126,127].

A randomized, double-blind clinical trial was conducted with 51 women with RA. The participants were divided into two groups: one group received a daily dose of 500 mg of quercetin, while the other group received a placebo for 8 weeks. At the end of the trial, there were no significant differences observed between the quercetin and placebo groups in terms of markers of oxidative stress, such as total antioxidant capacity, oxidized low-density lipoprotein, malondialdehyde (MDA), and CRP, as well as blood pressure [128].

In a separate study, 50 women diagnosed with RA were divided into two groups receiving a daily dose of 500 mg of quercetin or placebo for 8 weeks. The quercitin group had a decrease in early morning stiffness, morning pain, and discomfort following activity. Additionally, there was a reduction in plasma TNF-α levels as well as DAS-28 and HAQ scores [129].

Another clinical study was conducted to examine the impact of a combination of orally administered glucosamine-chondroitin-quercetin glucoside (GCQG) supplements on the synovial fluid characteristics of patients with OA and RA. Forty-six patients with OA and twenty-two patients with RA were given the GCQG supplement orally for 3 months. Various characteristics of the knee joints were observed and measured before and during the administration of supplements. The patients with OA experienced a notable enhancement in pain symptoms, daily activities such as walking and climbing stairs, and the visual analog scale. Additionally, there were observed alterations in the properties of the synovial fluid, including changes in protein concentration, the molecular size of hyaluronic acid, and the concentration of chondroitin-6-sulfate. Nevertheless, the RA patients did not exhibit any such outcomes. The results indicate that the GCQG supplement had a distinct impact on enhancing the characteristics of synovial fluid in patients with OA but not in RA patients [130].

In a randomized, placebo-controlled, double-blind clinical trial, 20 patients were randomly assigned to one of three groups to receive one of the following supplements: quercetin + vitamin C (166 mg + 133 mg per capsule), alpha-lipoic acid (300 mg per capsule), or a placebo for 4 weeks (3 capsules per day). Each treatment cycle consisted of 4 weeks, preceded by a wash-out interval of 2 weeks before the subject commenced the subsequent supplementing. The study found that administering dietary anti-oxidants at a dosage of 900 mg per day for 4 weeks did not result in any significant alterations in the blood biomarkers of inflammation (e.g., IL-1, IL-6, and TNF-α) or the severity of the disease in patients with RA who were undergoing conventional medical therapies [131].

There are currently no clinical trials evaluating the effects of quercitin treatment in RA patients (source: https://clinicaltrials.gov/search?cond=Rheumatoid%20Arthritis&intr=quercitin accessed on 3 April 2024).

### 4.5. Baicalin

Baicalin isolated from *Scutellaria radix* exhibited anti-inflammatory effects through the suppression of NF-κB-mediated expression, down-regulation of molecules responsible for leukocyte migration, immune cell adhesion, and signaling within inflamed tissues (intercellular adhesion molecule 1 (ICAM-1), and vascular cell adhesion protein 1 (VCAM-1)) [132], enzymes involved in pro-inflammatory response (COX-1, COX-2, inducible nitric oxide synthase (iNOS)), inflammatory mediators (IL-1β, IL-6, IL-17, and TNF-α) [132,133], metalloproteinases (MMP-2, MMP-9), autophagy-related proteins, and suppressed signaling through JAK1/STAT3 [134,135] and NF-Κb pathway [136]. Furthermore, in vivo, baicalin suppressed the expansion of Th17 cells in the spleen and inhibited the binding of lymphocytes to cultured synoviocytes that were induced by IL-17 [132].

The administration of baicalin (50 mg/kg) through intraperitoneal injection not only caused a decrease in body weight and the development of insulin resistance (HOMA-IR) in obese mice (C57BL/6J) but also led to a reduction in glucose intolerance and high blood sugar levels. This study also showed that baicalin did not exhibit hepatotoxicity in mice [137]. The structural conversion of baicalin to baicalein can occur through the activity of gut bacteria and associated enzymes, which can impact the effectiveness of baicalin. Only a minor fraction of baicalin is assimilated by the human body in its original form, while the majority of it undergoes hydrolysis into baicalein by bacteria and is subsequently absorbed by the body. Hence, it is important to take into account the impact of antibiotics on the pharmacokinetic characteristics of baicalin, since they can hinder the activity of gut bacteria when baicalin is administered in combination with antibiotics. The co-administration of baicalin has a consistent pharmacokinetic effect, wherein baicalin can considerably modify the pharmacokinetics of drugs that are metabolized by the same CYP enzyme or have high protein binding, as reviewed by Wen et al. [138]. Nevertheless, single oral dosages ranging from 100 to 2800 mg of baicalein demonstrated safety and good tolerance. There were no indications of liver or renal toxicity in the clinical laboratory examinations [139].

There is only one clinical trial investigating the impact of baicalin on lipid profiles and inflammatory responses in RA patients. A total of 374 patients diagnosed with both coronary artery disease and RA were randomly assigned to receive either 500 mg of baicalin or placebo orally daily for 12 weeks. After the research period, lipid profiles, cardiotrophin-1, high sensitivity CRP, and EULAR responses were measured. Baicalin demonstrated efficacy in lowering blood lipids and reducing inflammation in patients diagnosed with both coronary artery disease and RA, thereby providing support for its potential use in future clinical settings [140].

Table 1 provides a summary of pre-clinical and clinical studies related to the use of curcumin, bosewllic acids, resveratrol, quercitin, and baicalin in the treatment of RA disease.

### 4.6. Plant Extracts

When evaluating the use of whole plant extracts and isolated natural compounds for treating RA, it is necessary to consider the different benefits and disadvantages associated with their use. Whole plant extracts have the benefit of possessing a variety of chemicals that can work together to improve effectiveness, minimize side effects, and target several features of RA pathogenesis at the same time. However, challenges arise in standardizing and ensuring consistency in composition and potency between batches, as well as in identifying specific active compounds responsible for therapeutic effects. Isolated natural compounds, on the other hand, allow for precise control over purity and concentration, facilitating targeted therapy and reducing the risk of interactions with medications. Yet, the narrow focus of isolated compounds may overlook synergistic effects observed in whole plant extracts and limit the breadth of therapeutic benefits. Ultimately, the choice between whole plant extracts and isolated compounds depends on factors such as treatment goals, patient preferences, and available scientific evidence.

Advancements in extraction techniques, standardization methods, and quality control measures may address challenges associated with whole plant extracts, improving consistency and efficacy. Additionally, interdisciplinary research efforts combining traditional knowledge with modern scientific approaches may elucidate the mechanisms of action and optimize treatment protocols for both whole plant extracts and isolated natural compounds. Moreover, the growing interest in personalized medicine and integrative approaches to healthcare may lead to tailored treatment regimens that harness the synergistic effects of whole plant extracts while leveraging the precision and targeted therapy offered by isolated compounds. However, further research, including well-designed clinical trials and long-term safety studies, is needed to fully understand the therapeutic potential and establish the role of whole plant extracts and natural compounds in the comprehensive management of RA.

**Figure 6:**
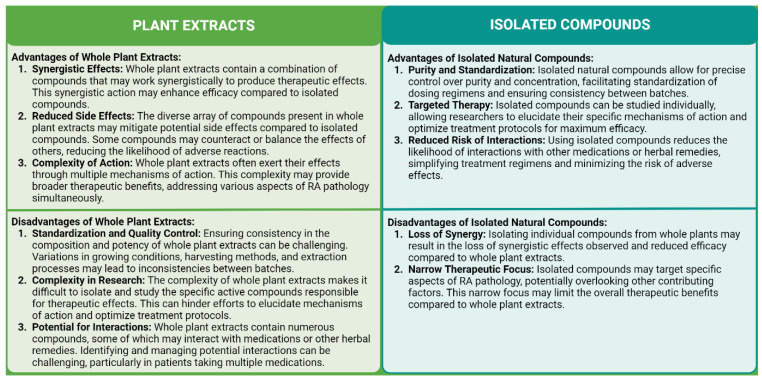
Advantages and disadvantages of the use of plant extracts and isolated compounds in the treatment of rheumatoid arthritis (RA).

Clinical investigations of plant extracts are pivotal in identifying promising natural compounds for drug development, particularly in diseases such as RA. These studies enable the identification of active components by correlating patients’ responses to treatment with the composition of the plant extract, leading to the isolation and characterization of specific compounds through rigorous chemical and biological analyses. By elucidating the mechanisms of action underlying the therapeutic effects of plant extracts, researchers can refine their search for active compounds and identify targets for drug development. Isolated natural compounds identified through clinical investigations undergo further optimization to enhance their pharmacological properties and advance to preclinical and clinical development stages. The importance of such studies lies in their potential to discover novel therapeutic agents derived from natural sources, offering opportunities to expand treatment options and address unmet medical needs in RA and other diseases (Figure 6).

A comparative analysis was conducted to assess the anti-oxidant capabilities of two polyherbal formulations, *Maharasnadhi quathar* (MRQ) and *Weldehi choornaya* (WC), which are utilized by Ayurvedic medical practitioners in Sri Lanka for treating patients with RA. The efficacy of these preparations was evaluated in patients based on the impact of the following factors: (a) the activity of anti-oxidant enzymes including SOD, GPX, and CAT; (b) the level of lipid peroxidation, measured by the generation of thiobarbituric acid reacting substances (TBARS); and (c) the concentrations of serum iron, hemoglobin, and the total iron binding capacity. The study’s findings indicate that MRQ has significantly higher anti-oxidant potential compared to WC. After being treated with MRQ for 3 months, the initial levels of plasma SOD, GPX, and catalase activities were increased by 44.6%, 39.8%, and 25.2%, respectively. Patients treated with WC for the same duration as MRQ did not experience any notable enhancement in any of these enzyme activities. The pharmacological preparations both decreased the degree of lipid peroxidation in the plasma of RA patients. However, the reduction achieved in 3 months by MRQ (34%) was significantly greater than that achieved by WC (21.8%). Treatment with MRQ, but not WC, significantly improved the total serum iron and Hb concentrations as well as TIBC in the RA patients included in the study. After a 3-month treatment with MRQ, the levels of total serum iron, hemoglobin, and TIBC in patients showed improvements of 26.8%, 24.8%, and 16.1%, respectively [141].

*Tripterygium wilfordii Hook F* (TWHF), commonly known as “*Thunder God Vine*”, has been studied for its potential therapeutic effects in RA. TWHF contains various bioactive compounds, including diterpenoids, triterpenoids, and alkaloids, which exhibit anti-inflammatory and immunosuppressive properties. Research on TWHF in RA has shown promising results in some studies. The first clinical trial on TWHF was performed in 2001, the efficacy and safety of ethyl acetate (EA) extracts were investigated. The study indicated that the EA of TWHF at dosages up to 570 mg/day demonstrated safety, while doses beyond 360 mg/day showed clinical efficacy in patients with RA [142]. In another prospective, double-blind, placebo-controlled trial including patients who failed conventional therapy treatments, subjects were randomized to receive either a placebo or a low-dose (180 mg/day) or high-dose (360 mg/day) extract for 20 weeks. This was then followed by the open-label extension period. Clinical responses were determined based on a 20% enhancement in disease activity as per the criteria established by the ACR. There were no instances of patients discontinuing participation due to adverse events throughout the open-label extension. The ethanol/ethyl acetate extract of TWHF showed therapeutic efficacy in individuals with RA who did not respond to conventional treatment. In this investigation, the TWHF extract was found to be well tolerated by the majority of patients when administered at therapeutic dosages [143]. A randomized controlled trial conducted in the United States compared the efficacy and safety of TWHF extract with sulfasalazine for the treatment of active RA. The study, involving 121 patients with RA and six or more painful and swollen joints, found that TWHF extract, administered at a dose of 60 mg three times daily, resulted in a significantly higher rate of achieving the ACR20 (20% improvement) compared to sulfasalazine over 24 weeks. Patients were allowed to continue continuous doses of oral prednisone or NSAIDs during the trial. While limitations included a dropout rate in both treatment groups, with only 62% and 41% of patients continuing TWHF extract and sulfasalazine, respectively, the study highlights the potential of TWHF extract as an effective treatment option for RA when used alongside standard therapies [144]. A multicenter, open-label, randomized controlled trial was performed to compare the effects of MTX treatment (12.5 mg once a week), TWHF (20 mg three times a day), or the two in combination. TWHF monotherapy was comparable to that of MTX monotherapy in managing disease activity in patients with active RA. Additionally, the combination of MTX and TWHF was found to be superior to MTX monotherapy in this regard [145]. Subsequent research has shown that the effectiveness of TWHF monotherapy is comparable to that of MTX monotherapy in managing disease activity and slowing down the progression of radiological damage in individuals with active RA over a 2-year treatment period [146].

*Uncaria tomentosa* (UT), commonly known as cat’s claw, is another herbal supplement that has been studied for its potential effects on RA. Cat’s claw is native to the Amazon rainforest and has a long history of traditional use for various health conditions, including arthritis. Research on UT in RA is somewhat limited compared to other herbal remedies, but some studies have suggested potential benefits. Cat’s claw contains several active compounds, such as alkaloids and polyphenols, which have anti-inflammatory and immunomodulatory properties. These compounds may help reduce inflammation and alleviate symptoms associated with RA. A total of forty patients who were receiving either sulfasalazine or hydroxychloroquine were included in a randomized study that lasted for 52 weeks and consisted of two phases. During the initial phase, which lasted for 24 weeks and followed a double-blind, placebo-controlled design, participants were administered either UT extract or a placebo. All patients were administered the plant extract throughout the second phase, which lasted for 28 weeks. The administration of the UT extract for twenty-four weeks led to a decrease in the number of sore joints in comparison to the placebo. Patients who were administered the UT extract exclusively during the second phase observed a decrease in the quantity of painful and swollen joints in comparison to the measurements taken after 24 weeks of receiving a placebo. Only negligible adverse effects were detected. This pilot investigation provided evidence of the relative safety and efficacy of the extract from the pentacyclic chemotype of UT in patients receiving sulfasalazine or hydroxychloroquine [147].

Consuming pomegranate (*Punica granatum*) extract has been demonstrated to decrease the occurrence and intensity of CIA in rats. In a pilot clinical study, pomegranate consumption decreased DAS28 in patients with RA, and this benefit could be attributed to the anti-oxidative nature of pomegranates. As a consequence, supplementing the diet with pomegranates may be a beneficial supplemental approach to reduce the severity of clinical symptoms in RA patients [148]. In another clinical study, a total of 55 patients with RA were randomly divided into two groups: an intervention group consisting of 30 patients and a control group consisting of 25 patients. The intervention group was administered two capsules containing 250 mg of pomegranate extract daily for 8 weeks, while the control group received two capsules containing 250 mg of cellulose. At the start and after 8 weeks of the study, participants completed the HAQ and DAS28. Additionally, serum concentrations of CRP, MMP3, MDA), GPX, and ESR were analyzed. Pomegranate extract relieved disease activity and improved some blood biomarkers of inflammation and oxidative stress in RA patients [149].

Gingerol, a bioactive constituent present in *Zingiber officinale*, commonly known as ginger, is a frequently employed spice and medicinal plant. The aforementioned substance is a highly aromatic phenolic compound that is classified as gingerol, belonging to a family of structurally similar compounds. Gingerol can be described as a phenolic compound that features a hydroxyl functional group covalently bonded to an extended hydrocarbon chain. The predominant form of gingerol is found in unadulterated fresh ginger, and its levels are subject to variation based on the type of ginger and the environmental factors during cultivation. The potential health benefits of gingerol have been the subject of scientific investigation, with particular attention paid to its anti-oxidant, anti-inflammatory, and anti-emetic properties. Apart from gingerol, ginger comprises other associated compounds such as shogaols and paradols that are produced during the process of drying or cooking ginger. The aforementioned compounds are known to play a role in the collective bioactivity exhibited by ginger [150]. A total of seventy women diagnosed with RA took part in a randomized, double-blind, placebo-controlled, parallel-design trial. The participants were randomly allocated into two cohorts, with one group receiving two tablets containing 500 mg of garlic extract and the other group receiving two placebo tablets daily for 8 weeks. At the beginning and conclusion of week 8, the authors of the study measured the levels of total anti-oxidant capacity and MDA in the serum, as well as assessed the quality of life using HAQ. The results indicate that taking garlic supplements for 8 weeks led to notable enhancements in oxidative stress and HAQ in women diagnosed with RA [151].

*Stephania tetrandra*, also known as Han Fang Ji or Fen Fang Ji, is a medicinal plant used in traditional Chinese medicine that has been investigated for its potential therapeutic effects on RA. *Stephania tetrandra* contains various bioactive compounds, including alkaloids, flavonoids, and lignans, which have been studied for their anti-inflammatory and immunomodulatory properties. Some research suggests that these compounds may help reduce inflammation and alleviate symptoms associated with RA. Following a 12-week treatment with *Stephania tetrandra* extract, there was a notable reduction in both the proportion and count of granulocytes in the peripheral blood. The levels of lipid peroxide and human granulocyte elastase in plasma decreased considerably. Moreover, both the leukocyte/elastase ratio and the granulocyte/elastase ratio showed a considerable rise. The results of this study indicate that administering *Stephania tetrandra* extract has a suppressive impact on excessive activation of granulocytes, leading to an improvement in inflammation associated with RA [152].

Cannabinoids, the active compounds in cannabis, including tetrahydrocannabinol (THC) and cannabidiol (CBD), have been shown to have analgesic properties. They can modulate pain perception and reduce inflammatory pain, potentially providing relief for RA-related pain. Both THC and CBD have demonstrated anti-inflammatory properties in preclinical studies. They can reduce the production of pro-inflammatory cytokines and inhibit immune cell activation, which are key factors in the pathogenesis of RA. Cannabis-based medicine (Sativex) was evaluated in a randomized, double-blind, study in 58 patients over 5 weeks of treatment. Sativex was delivered via oromucosal spray in the evening, and evaluations were conducted the next morning. The efficacy outcomes evaluated included pain during movement, pain at rest, morning stiffness, and sleep quality using a numerical rating scale, the short-form McGill pain questionnaire, and the DAS28 measure of disease activity. Notably, Sativex medication resulted in a considerable reduction in pain and a significant decrease in disease activity. Although the changes were minor and varied throughout the population, they were significant in terms of therapeutic advantages and highlighted the necessity for further thorough exploration of this specific indication [153].

*Andrographis paniculata*, commonly known as “*Andrographis*”, is a medicinal herb that has been used in traditional medicine systems, particularly in Ayurveda and traditional Chinese medicine. It has gained attention for its potential therapeutic effects on various health conditions, including RA. Andrographis contains bioactive compounds such as andrographolides, flavonoids, and diterpenoids, which have demonstrated anti-inflammatory, immunomodulatory, and anti-oxidant properties in preclinical studies. These compounds may help reduce inflammation and alleviate symptoms associated with RA. A prospective, randomized, double-blind, and placebo-controlled study was conducted on individuals with RA. Tablets containing Paractin, an extract derived from *A*. *paniculata* with a concentration of 30% total andrographolides, were given to 60 patients with active RA. The tablets were taken three times a day for 14 weeks, followed by 2 weeks without taking any medication. The main results were the measurement of pain intensity using a horizontal visual analog pain scale (VAPS). Furthermore, the clinical parameters of ACR, EULAR, and SF36 were documented. The active group saw a reduction in joint pain, swelling, and tenderness compared to the placebo group by the end of the treatment. However, the pain differences did not reach statistical significance [154].

López Mantecón et al. investigated the activity and safety of *Mangifera indica* extract (Vimang tablets, 300 mg) used together with methotrexate (MTX) in RA patients. Twenty patients diagnosed with active RA received a year-long treatment consisting of MTX at a dosage of 12.5 mg per week, along with NSAIDs and/or prednisone at a dosage of 5–10 mg per day. The experimental group received a daily supplementation of 900 mg of extract, while the control group, continued with their usual treatment for 180 days. The assessment of RA activity involved the examination of sore and swollen joints, and the measurement of ESR, DAS 28, VAS, and HAQ. The effectiveness of the treatment was demonstrated using the ACR criteria. Statistically significant improvement in DAS 28 metrics was only observed in the patients in the MTX-Vimang group, compared to their baseline data. However, no differences were found between the groups. The ACR improvements reached a significant 80% only in the MTX-Vimang group after 90 days. In the MTX-Vimang group, all patients had a reduction in the use of NSAIDs, and 70% of them eliminated gastrointestinal adverse effects caused by the previous treatment [155].

*Artemisia annua*, also known as sweet wormwood or Qinghao, is a plant with a long history in traditional Chinese medicine. It is best known as the source of artemisinin, a compound used to treat malaria. However, *Artemisia annua* has also been investigated for its potential anti-inflammatory and immunomodulatory effects, which may be relevant in the context of RA [156,157]. A randomized controlled trial showed that the combination of *Artemisia annua* L. extract with methotrexate and leflunomide demonstrated superior efficacy and safety compared to the standard treatment of methotrexate and leflunomide alone for active RA [158]. In the pilot randomized, placebo-controlled clinical trial evaluating the effectiveness and safety of an extract derived from *Artemisia annua* provided for 12 weeks to evaluate its ability to alleviate pain, stiffness, and functional limitations caused by osteoarthritis in the hip and knee joints. Treatment of patients with *Artemisia annua* (150 mg/twice daily) resulted in clinically relevant reductions in pain over 12 weeks [159].

*Berberis vulgaris*, or a similar species, has been used in traditional medicine for its various purported health benefits. While most commonly associated with its use in treating digestive and other ailments, some compounds found in barberry plants, notably berberine, have been investigated for their potential anti-inflammatory and immunomodulatory effects, as recently reviewed by other authors [160]. One of the clinical trials investigated the immunomodulatory effect of the hydroalcoholic extract of black barberry on immunological mediators in individuals with active RA. During randomized, double-blind, placebo-controlled clinical research, women with RA were administered two capsules each day for 12 weeks. Each capsule contained either 1000 mg of black barberry extract or a placebo made of maltodextrin. Demographic indicators, physical activity, nutritional consumption, and disease activity were examined using appropriate questionnaires. The levels of cytokines IL-2, IL-4, IL-10, and IL-17 in the blood sample were quantified using peripheral blood. Initially, there were no disparities between the two groups in terms of demographic factors, physical activity levels, or nutritional consumption. Supplementing with black barberry decreased the intensity of RA. The intervention had no significant impact on IL-2 and IL-4 cytokines. However, IL-17 levels decreased considerably in the black barberry group after the intervention, whereas IL-10 showed a considerable increase in this group. The study determined that Barberry extract has the potential to decrease inflammation and enhance the production of anti-inflammatory cytokines in individuals with RA. Additionally, it was found that Barberry extract can boost the immune response by raising the production of Th2 cells [161].

*Nigella sativa*, commonly known as black seed or black cumin, is a flowering plant native to South Asia and the Middle East. Its seeds have been used in traditional medicine for centuries, and modern research has explored its potential therapeutic effects in various conditions, including RA. *Nigella sativa* seeds contain several bioactive compounds, with thymoquinone being one of the most studied. Thymoquinone is known for its anti-inflammatory, anti-oxidant, and immunomodulatory properties [162,163]. In the first clinical trial, the treatment of female RA patients (*n* = 40) with *Nigella sativa* capsules before and after placebo administration resulted in a reduction in DAS28, the number of swollen joints, and the duration of morning stiffness. After taking the preparation, 42.5% of the patients showed a significant improvement in disease activity according to the ACR20 response criteria, whereas 30% showed improvement according to the EULAR response criteria [164]. In another randomized, double-blinded placebo-controlled clinical trial, 43 female RA patients were administered 1 g of *Nigella sativa* or a starch capsule (placebo) in two divided doses. The study evaluated DAS28 as well as the abundance of CD4+, CD8(+), and CD4(+)CD25(+) T cells. Administration of *Nigella sativa* capsules resulted in a notable decrease in the serum high-sensitivity C-reactive protein (hs-CRP) level and DAS-28 score, as well as an enhanced number of swollen joints when compared to the baseline and placebo groups. There was a similar CD4(+) T cell percentage found in both the *Nigella sativa*-treated group and the placebo group at both the beginning and end of the therapy. The treatment with *Nigella sativa* led to a decrease in the percentage of CD8(+) T cells and an increase in the percentage of CD4(+)CD25(+) T cells, as well as an increase in the CD4(+)/CD8(+) ratio when compared to both the placebo and the start of the study. In the *Nigella sativa* group, there was a noteworthy negative association between changes in CD8(+) and changes in CD4(+)CD25(+) T cells, as well as a substantial positive correlation between changes in CD4(+)CD25(+) T cells and changes in the CD4(+)/CD8(+) ratio [165]. The subsequent randomized, double-blind, placebo-controlled clinical trial assessed the anti-inflammatory and anti-oxidant characteristics of *Nigella sativa* oil in individuals diagnosed with RA. The intervention group participants were administered two capsules of *Nigella sativa* oil, each containing 500 mg of the natural compound, daily for 8 weeks. The second group ingested two placebo pills daily for the same duration. Baseline and end-of-trial measurements were taken for serum levels of TNF-α, IL-10, and oxidative stress markers in whole blood. The concentration of IL-10 in the *Nigella sativa* group showed an increase, while a notable decrease in serum MDA and NO levels was observed compared to the initial measurements. There were no notable disparities in the levels of TNF-α, SOD, and CAT [166].

Willow bark, derived from various species of the willow tree (genus *Salix*), has been used for centuries as a natural remedy for pain and inflammation. The primary active ingredient in willow bark is salicin, a compound chemically similar to acetylsalicylic acid. It is believed that this compound and others in willow bark have anti-inflammatory and analgesic effects, which have prompted interest in its use for conditions such as RA and OA. Biegert et al. evaluated the effectiveness and safety of a standardized extract derived from willow bark in individuals diagnosed with OA and rheumatoid arthritis. However, the results of the study indicated no positive effects of the extract on the WOMAC pain score or VAS [167].

Table 2 provides a summary of the clinical use of plant extracts in the treatment of RA disease.

## 5. Other Compounds

### 5.1. Apigenin

Apigenin represents another flavonoid compound with anti-oxidative and anti-inflammatory properties [168]. Specifically in RA, it was shown to inhibit proliferation [169] and induce the apoptosis of FLS through the ERK1/2 pathway [169,170,171,172]. In contrast, its anti-inflammatory activity stems from the reduced production of inflammatory mediators such as IL-1β, IL-6, and TNF-α [172,173,174,175], partially through the inhibition of the COX2 enzyme [176]. Apigenin showed inhibitory effects on collagenase activity and effectively decreased LPS-induced NO production in RAW264.7 macrophage cells. Apigenin pretreatment also reduced the expression of COX-2, VCAM-1, ICAM-1, and E-selectin induced by either LPS or TNF-α [177]. Apigenin has also been found to inhibit angiogenesis (through down-regulation of vascular endothelial growth factor (VEGF) and vascular endothelial growth factor receptor (VEGFR)) and osteoclastogensis through the suppression of the RANKL/osteoprotegerin pathway in a CIA mouse model [169].

### 5.2. Betulinic Acid

Betulinic acid (BA) is a naturally occurring triterpenoid compound found in various plants, including the bark of white birch trees (*Betula alba*) and other plant species. It has gained attention for its diverse pharmacological properties, including anti-inflammatory, anti-oxidant, anti-cancer, anti-viral, and immunomodulatory effects [178].

It was found that BA suppressed the growth, motion, and infiltration of FLSs and reduced the effects of TNF-α exposure, the expression of MMPs, and the production of inflammatory cytokines (IL-6, IL-8) in FLS. In addition, BA inhibited the activation of the AKT/NF-κB pathway in TNF-α-exposed FLS [179]. Similarly, BA treatment inhibited the migration, invasion, and rearrangement of the actin cytoskeleton in RA FLSs. Furthermore, it was discovered that the mRNA levels of IL-1β, IL-6, IL-8, and IL-17A were significantly reduced when TNF-α-induced RA FLSs were treated with BA. From a mechanistic standpoint, the administration of BA resulted in a reduction in the activation of the NF-κB signal pathway and the accumulation of NF-κB in the nucleus, which ultimately led to a decrease in synovial inflammation and joint destruction in mice with CIA [180].

BA effectively lowered hind paw swelling, synovial tissue proliferation, cartilage degeneration, vasospasm, and blood levels of IL-1β, IL-6, and TNF-α in rats with CIA. BA effectively suppressed the growth of fibroblast-such as synoviocytes that were exposed to TNF-α treatment. Additionally, BA also reduced the release of IL-1β and IL-6, suppressed the transcription of VEGF and transforming growth factor β (TGF-β), and reduced the expression of the NF-ĸB pathway proteins (NF-kB-P65, IkBα, and IKKα/β) in TNF-α-stimulated FLS [181].

### 5.3. Curculigo Glycoside

Curculigo glycoside is a bioactive compound derived from the rhizomes of the *Curculigo orchioides* plant, which is native to tropical regions of Asia, including India and China. This plant has a long history of use in traditional medicine, particularly in Ayurveda and traditional Chinese medicine, where it is valued for its purported aphrodisiac, adaptogenic, and tonic properties. It is a type of steroidal saponin, which is a class of natural compounds found in plants that have diverse pharmacological activities. It has been identified as one of the major bioactive constituents responsible for the medicinal properties of *Curculigo orchioides*. Research on curculigo glycoside and its potential health benefits is still in its early stages, but preliminary studies suggest that it may have several pharmacological effects [182,183].

Specifically in the treatment of RA, curculigoside A (CA) was shown to alleviate arthritis symptoms in rats with adjuvant-induced arthritis. This effect was likely achieved by decreasing the levels of pro-inflammatory molecules (IL-6, IL-1β, TNF-α) and PGE2 [184]. It was also found that CA improved the symptoms of arthritis in rats with type II CIA and decreased the levels of inflammatory markers, including TNF-α, IL-1β, IL-6, IL-12, and IL-17A. The potential molecular mechanism by which it protects against arthritis may be associated with the JAK/STAT/NF-κB signaling pathway [185].

Two network pharmacology studies revealed that CA likely induced anti-osteoporosis and anti-RA actions by modulating specific biological pathways, namely nitrogen metabolism, the estrogen signaling system, the RAP1 signaling pathway, and the PI3K/AKT signaling pathway. The qPCR results demonstrated that pretreatment of RAW264.7 cells with CA led to a significant reduction in the expression of epidermal growth factor receptor (EGFR), dual-specificity mitogen-activated protein kinase kinase 1 (MEK1), MMP2, fibroblast growth factor receptor 1 (FGFR1), and myeloid cell leukemia 1 (MCL1) encoding genes [186].

Treatment with *Curculigo orchioide* extract in the rodent model of arthritis had significant inhibitory effects on inflammatory cell infiltration, pannus formation, and cartilage damage. It also suppressed the expression of MMP9 in rats with CIA, reduced JUN protein expression in the ankle joints, and downregulated the expression of PTGS2 [187].

### 5.4. Epigallocatechin Gallate

Epigallocatechin gallate (EGCG) is a catechin compound belonging to the class of flavonoids and is known as a prevalent and biologically active compound found in green tea. EGCG is renowned for its powerful anti-oxidant properties, which aid in protecting cells and tissues against ROS. The anti-oxidant action of EGCG is due to its capacity to eliminate free radicals and regulate the activity of anti-oxidant enzymes [188]. Its use in RA holds several potential advantages. For example, EGCG has been shown to inhibit the production of pro-inflammatory cytokines and chemokines, such as IL-1β, TNF-α [189], and IL-6 [190], which are known to play key roles in the pathogenesis of RA. By reducing inflammation, EGCG may help alleviate symptoms such as joint pain, swelling, and stiffness associated with RA [191,192].

Oxidative stress is implicated in the progression of RA, contributing to tissue damage and inflammation in the joints. EGCG’s potent anti-oxidant properties help neutralize free radicals and ROS, thereby protecting cells and tissues from oxidative damage. This antioxidant activity may help mitigate joint damage and slow the progression of RA. In the study utilizing the CIA model in Wistar albino rats, the groups that received EGCG showed improvements in blood levels of TNF-α, IL-17, and MDA, as well as elevated levels of SOD, CAT, GPX, and the expressions of NRF2 and heme oxygenase-1 (HO-1). Furthermore, histological examination has revealed a decrease in perisynovial inflammation and cartilage-bone degradation in the groups that were supplied with EGCG. The findings indicate that EGCG exerts an anti-arthritic impact by modulating the equilibrium between oxidative and anti-oxidant processes, as well as the levels of cytokines, in the RA model [193].

EGCG can modulate the activity of immune cells involved in the pathogenesis of RA, such as T cells, B cells, and macrophages. It may help regulate the balance between pro-inflammatory and anti-inflammatory immune responses, leading to a dampened immune response and reduced inflammation in RA [194]. For example, EGCG improved the condition of arthritic mice by stimulating the development of indoleamine-2,3-dioxygenase-producing dendritic cells, enhancing the presence of T regs, and activating the NRF-2 anti-oxidant pathway [195].

EGCG has been shown to inhibit the activity of enzymes involved in the degradation of cartilage and bone, such as MMPs and cathepsins [189,196,197]. Moreover, EGCG inhibited the formation of osteoclasts and improved the symptoms of experimental arthritis in rodents [197,198]. In another study, EGCG enhanced PI3-kinase activity, leading to the stimulation of AKT and RAF-1 and a decrease in AP-1 binding to the monocyte chemoattractant protein-1 (CCL2) promoter, ultimately resulting in reduced CCL2 synthesis in osteoblasts. CCL2 is a chemokine involved in the regulation of immune cell migration and inflammation. CCL2 plays a critical role in the recruitment of monocytes, memory T cells, and dendritic cells to sites of inflammation and tissue injury. It acts as a chemoattractant, guiding immune cells to the site of inflammation. EGCG mitigates the intensity of CIA, likely by inhibiting CCL2 production in osteoblasts to reduce macrophage infiltration [199]. The treatment with EGCG reduced the symptoms of CIA in mice, prevented the formation of osteoclasts and the activation of T helper 17 cells, and increased the population of regulatory T cells. The anti-arthritic actions of EGCG at the molecular level may result from the activation of ERK, NRF2, and HO-1, as well as the suppression of STAT3 activation [200]. The resistance of RA synovial fibroblasts to TNF-α-induced apoptosis is primarily caused by the overexpression of the anti-apoptotic protein MCL-1. EGCG was shown to trigger apoptosis and enhance the susceptibility of RA synovial fibroblasts to TNF-α-induced death by specifically inhibiting MCL-1 expression. Therefore, it has been proposed as a potential supplementary therapy for controlling the invasive expansion of synovial fibroblasts in RA [201].

Despite the rising enthusiasm for EGCG as a complementary therapeutic approach in RA treatment, it has relatively poor bioavailability, meaning that only a small fraction of ingested EGCG is absorbed into the bloodstream and reaches target tissues. This limited bioavailability may affect the effectiveness of EGCG as a therapeutic agent in RA. Interestingly, the preliminary studies suggest that it is possible to strengthen the articular cartilage by direct injection of EGCG into the affected RA joints, bypassing the bioavailability burden [202]. The second option involves the development of novel formulations or delivery systems that enhance the bioavailability of EGCG. For example, encapsulating EGCG in nanoparticles or liposomes could improve its stability and absorption [188]. Song et al. developed macrophage cell membrane-camouflaged nanoparticles (M-EC), which were composed of EGCG and cerium (IV) ions. The M-EC exhibited scavenging efficacy towards several forms of ROS and reactive nitrogen species (RNS) due to its geometric resemblance to the active metal sites of natural anti-oxidant enzymes. The macrophage cell membrane facilitated the evasion of M-EC from the immune system, as well as their uptake by inflammatory cells and particular binding to IL-1β. Following the administration of M-EC through tail vein injection in the CIA mice, the M-EC cells gathered at the inflamed joints and successfully repaired the bone erosion and cartilage loss associated with RA. This was accompanied by the reduction of synovial inflammation and the prevention of further cartilage erosion [203]. In another study, EGCG was combined with extracellular vesicles derived from macrophages, which resulted in a decrease in the expression of apoptosis-related proteins and upregulation of type II collagen expression. EGCG exhibited anti-inflammatory effects by reducing edema, decreasing synovial hyperplasia, repairing cartilage, and mitigating arthritis-related pathology scores in rats with arthritis [204]. Lee et al. constructed conjugates of hyaluronic acid and EGCG (HA-EGCG). These conjugates were able to specifically target fibroblast-like synoviocytes by interacting with HA-CD44. Under simulated physiological conditions, these conjugates demonstrated greater anti-proliferative and anti-inflammatory effects compared to EGCG. The use of near-infrared fluorescence imaging demonstrated the selective buildup of the conjugates in inflamed joints of a rat model with CIA [205].

The therapeutic effects of EGCG may be dose-dependent, meaning that higher oral doses may be required to achieve meaningful clinical benefits. However, higher doses of EGCG may also increase the risk of adverse effects or interactions with medications. Surprisingly, it was found that EGCG can ameliorate the hepatotoxicity of MTX. The administration of MTX resulted in a substantial elevation in aspartate aminotransferase, alanine transaminase, alkaline phosphatase, and lactate dehydrogenase concentrations in the plasma. However, this effect was effectively counteracted by prior treatment with EGCG. In addition, the hepatic oxidative stress caused by MTX, as indicated by the increased production of MPO, elevated protein carbonylation, lipid peroxidation, and reduced activity of anti-oxidant enzymes, was alleviated by prior administration of EGCG. The introduction of an anti-oxidant, such as EGCG, during continuous administration of MTX improves the condition of MTX-induced hepatotoxicity [206]. Therefore, combinational treatment with standard therapies and natural compounds such as EGCG could protect normal cells from their toxic effects.

Conducting well-designed clinical trials to evaluate the safety, efficacy, and optimal dosing of EGCG in RA patients is essential for establishing its role as a therapeutic agent. Rigorous clinical research can provide valuable insights into the potential benefits and limitations of EGCG in RA management.

### 5.5. Icarin

Icarin and its metabolite are flavonoid glycosides isolated from *Epimedium grandiflorum,* known for their anti-oxidant, anti-inflammatory, and immunoregulatory properties [207]. It is mainly metabolized into icaritin [207]. Its role in the prevention and management of RA is not fully understood; however, preclinical evidence showed that this compound could positively affect RA via the reduced expression of β3 integrin, cathepsin K [208] and RANKL [209,210], reduce the number of Th17 cells [211], suppress MAPK or the NF-κB pathway, and alleviate bone degradation in the rodent models of CIA [212,213]. Its effect on FLS cells is also noteworthy. Icarin was shown to suppress the proliferation of these cells and promote their apoptosis [212,214,215]. Furthermore, it suppressed the secretion of TNF-α, IL-1β, and IL-6, thereby exerting anti-inflammatory activity [212,214].

### 5.6. Roburic Acid

Roburic acid (RBA) is a compound that belongs to the group of tetracyclic triterpenoids and is derived from terpenes. It is formed by the coupling of five-carbon isoprene units. Galls, which are abnormal growths resulting from insects feeding on plant tissues, were the initial source of isolation for this compound. *Gentiana macrophylla* and *Gentiana dahurica*, which are utilized in traditional Chinese herbal therapy, were also discovered to contain it. Initial research on this molecule revealed its extensive range of biological effects, such as its ability to operate as an anti-oxidant, anti-microbial, anti-atherosclerotic, and anti-inflammatory agent [178]. The anti-inflammatory properties of this compound stem from (a) the inhibitory effect on microsomal prostaglandin E2 synthase-1 (mPGES-1) [216] or (b) COX1/2 enzymes [217]; (c) suppression of the MAPK pathway and NF-κB translocation to the nucleus, leading to suppression of NO and IL-6 production in vitro [218]. Furthermore, it has been demonstrated that the suppression of NF-κB signaling is a result of the direct contact between RBA and TNF-α. This interaction prevents the natural ligand from binding to the tumor necrosis factor receptor 1 (TNF-R1) [219].

RBA was shown to suppress the development of osteoclasts derived from bone marrow macrophages without causing any cellular damage. Remarkably, the compound did not have any impact on osteoblastogenesis in a laboratory setting. In addition, RBA reduced the production of F-actin rings, the resorption of hydroxyapatite, and the expression of genes in osteoclasts and inhibited the activity of TNF receptor-associated factor 6 (TRAF6), which is an important protein that acts as an adapter following the interaction of RANKL-RANK, leading to the inhibition of NF-κB activity, reduced phosphorylation of ERK, and calcium oscillations. Conversely, RBA increased the activity of the anti-oxidative response element (ARE) and the protein levels of HO-1. These changes in the early stages ultimately resulted in the inhibition of the nuclear factor of activated T cell 1 (NFATc1) function and the production of proteins that play a role in the formation of osteoclasts and the breakdown of bone tissue. In addition, the therapeutic effects of RBA against bone loss were observed utilizing an ovariectomized mouse model [220]. Jia et al. developed a drug delivery system called RBA-NPs that responds to changes in pH and targets both CD44 and folate receptors. In the rat model of RA, the nanocarriers successfully transported RBA to regions of inflammation, resulting in a considerable improvement in therapeutic results compared to the unencapsulated form of RBA. Additionally, the nanocarriers greatly reduced levels of inflammatory cytokines and facilitated tissue healing. It was also demonstrated that M1 macrophages in the joints underwent reprogramming to the M2 phenotype via RBA. The reprogramming of macrophages, which affects the balance between pro- and anti-inflammatory cells, is likely responsible for the beneficial effect of this compound in RA by preserving immunological balance and limiting excessive inflammation. In addition, it was demonstrated that RBA-NPs induced a transition from the M1 to M2 phenotype by reducing the glycolysis level through inhibition of the ERK pathway [221].

Pre-clinical studies investigating apigenin, betulinic acid, curculigo glycoside, EGCG, icarin, and RBA use in the RA treatment were presented in Table 3.

## 6. Marketed Formulations

In recent years, there has been a notable increase in the number of marketed formulations derived from natural compounds and plant extracts, designed to address various aspects of RA. These formulations, which encompass a range of botanical products, herbal extracts, and isolated natural compounds, aim to provide therapeutic benefits while minimizing the adverse effects often associated with conventional pharmacological treatments. Commonly available products include capsules, tablets, topical creams, and teas, each containing a variety of active ingredients purported to have anti-inflammatory, analgesic, or immunomodulatory effects. For example, formulations containing turmeric (curcumin) and *Boswellia serrata* are marketed for their anti-inflammatory properties, while those with black cumin (*Nigella sativa*) and willow bark are valued for their analgesic effects. Despite the growing popularity of these products, there are significant challenges related to quality control, standardization, and bioavailability. Variability in the source and preparation methods can lead to inconsistencies in the concentration and efficacy of the active compounds. Additionally, many of these naturally derived substances suffer from poor bioavailability, which can limit their therapeutic impact. The emergence of these natural products in the commercial sector underscores the need for comprehensive clinical studies to validate their efficacy and safety, while also establishing guidelines for their optimal use in the management of rheumatoid arthritis.

## 7. Conclusions and Future Prospects

RA is a multifaceted autoimmune disorder that is characterized by persistent inflammation and degeneration of the joints. The limitations of conventional therapies for RA have prompted a surge of interest in investigating alternative approaches. Plant-derived natural compounds hold great promise for the treatment of RA owing to their potential anti-inflammatory, immunomodulatory, and analgesic properties. The present review provides a comprehensive analysis of the potential therapeutic activities of naturally extracted compounds and plant extracts in the management of RA. Although preclinical and clinical studies have yielded promising results, there are several challenges that must be overcome before naturally extracted compounds and extracts can be effectively integrated into standard clinical practice.

To optimize the utilization of naturally derived compounds in the treatment of RA, it is crucial to acknowledge and tackle a range of obstacles and constraints. The limited bioavailability of these compounds remains a significant concern. The investigation of strategies to improve the absorption, stability, and pharmacokinetic characteristics of natural substances is of utmost importance. The application of innovative drug delivery systems, such as nanocarriers and liposomes, offers a potentially fruitful approach to tackle the issues related to limited bioavailability and enhance the targeted delivery of these substances to affected joints.

The integration of naturally derived compounds with conventional therapeutic approaches exhibits the potential for enhanced outcomes in the management of the disease. There is a possibility that naturally occurring compounds possess the ability to augment the effectiveness of current therapies while mitigating their adverse reactions. The attainment of synergistic effects can be facilitated through meticulous formulation or combination strategies and the identification of dosing regimens that are optimal.

The effectiveness of RA therapy is influenced by various factors, such as gender, comorbidities, drug pharmacokinetics and pharmacodynamics, other pharmacological therapies, and drug interactions. Although current therapeutic methods do not incorporate routine monitoring, increasing evidence suggests the potential predictive value of individual biomarkers to personalize therapy and predict treatment response. The key to personalizing RA therapy is specific genetic variants. Gene polymorphisms involved in the metabolism and transport of DMARDs, i.e., MTX, can significantly impact its efficacy and toxicity. Specific polymorphisms in the *MTHFR* gene, which encodes 5,10-methylenetetrahydrofolate reductase, crucial in folate metabolism, can reduce enzyme activity [222]. The presence of the T allele in the C677T polymorphism (rs1801133) can decrease enzyme activity by 30–65%, leading to elevated homocysteine levels and an increased risk of drug toxicity [223,224] Other examples of genetic variants involve transporter genes that affect MTX availability, toxicity, or sensitivity. Predictive models that integrate genetic polymorphism information and their influence on drug efficacy or toxicity facilitate selecting appropriate doses and treatment regimens for individual patients, minimizing adverse effects and improving treatment effectiveness.

Interindividual variability in the pharmacokinetics and pharmacodynamics of DMARDs used in RA therapy is also crucial for safety and individual treatment responses. Drug pharmacokinetics is focused on drug absorption, distribution, metabolism, and elimination, which are influenced by factors such as age, gender, drug interactions, and the functions of organs responsible for drug elimination and detoxification. RA predominantly affects women more than men, partly due to the role of sex hormones in the immune response [225]. Therefore, consideration of gender-specific factors such as differences in body mass, hormone levels, and organ function is essential. Age may be correlated with the ability to metabolize and eliminate drugs, which can lead to variations in drug concentrations in the body and associated toxicity risks. Comorbidities such as kidney or liver impairment affect the elimination capacity of the drug and its pharmacodynamic effects. Patients with impaired renal function exhibit higher concentrations of MTX, increasing the risk of adverse effects [224]. Polypharmacy, the concurrent use of multiple drugs, also poses a risk due to pharmacokinetic interactions. For example, drugs that alter stomach pH can affect DMARD absorption, while liver enzyme inhibitors can alter its metabolism and efficacy [226]. Changes in serum protein concentrations can influence drug binding and biological availability. Patients with low albumin levels may experience higher levels of unbound drugs, increasing the risk of adverse effects [227]. Combining conventional drugs for RA with natural substances requires not only extensive knowledge of their properties and possible interactions but also detailed monitoring of the patient’s condition by the doctor throughout the entire treatment period. Natural compounds in the form of adjuvant therapy or additional supplementation may affect the pharmacokinetics and pharmacodynamics of drugs. Some substances of plant origin may act as inhibitors or inducers of cytochrome P450 (CYP450) enzymes, which translates into the concentration of drugs in the body. Additionally, natural compounds may have toxic properties that, when combined with conventional drugs, may increase the risk of side effects or toxicity profiles. If a natural substance has hepatotoxic properties, it will potentially damage the liver, and its effect may be increased by the cumulative use of hepatotoxic DMARDs. On the other hand, substances with anti-inflammatory properties may potentially enhance the effects of anti-inflammatory drugs used in RA [228,229,230].

Another consideration is the development of autoantibodies characteristic of autoimmune diseases. Their diagnosis and monitoring help predict the response to treatment and identify patients who need more intensive therapy. In RA, the principal autoantibodies include RF, ACPAs, and anti-drug antibodies (ADA) [222]. RF targets the Fc fragment of immunoglobulin G (IgG) and may be correlated with aggressive disease progression and poorer outcomes. Its clinical usefulness is limited due to its occurrence in healthy people and people with other autoimmune and/or non-autoimmune diseases [222,231]. In general, elevated RF levels may indicate a greater risk of DMARD treatment failure, necessitating the use of biologic drugs. ACPAs are autoantibodies against citrullinated proteins that occur in 70–90% of patients with RA and are characterized by high specificity (90–95%) [222,232]. ACPAs are produced locally in joints, where inflammatory processes lead to protein citrullination, which activates a complex immune response and the formation of ACPAs. High levels of ACPA may predict a poor response to traditional DMARD treatment, suggesting the need for alternative therapies. ACPA is also a good predictive marker of the disease. Approximately half of RA patients showed an abnormal serological profile even before the first symptoms appeared [233]. The use of biologic DMARDs often induces ADA, which diminishes therapeutic efficacy by reducing serum concentrations or neutralizing drug effects, resulting in clinical non-response. The mechanisms for ADA formation remain unclear but are associated with the immunogenicity of administered substances. For instance, ADA occurrence has been observed in nearly half of infliximab-treated patients and approximately 30% of those treated with adalimumab [222,234,235]. On the contrary, ADA is detected in only about 7% of patients treated with the less immunogenic etanercept [222]. Monitoring autoantibody levels demonstrates significant predictive potential, helping optimize therapy, identifying patients at risk of treatment failure, and adjusting therapeutic strategies. Future research may lead to these markers becoming standards for monitoring and personalizing RA therapy.

**Figure 7:**
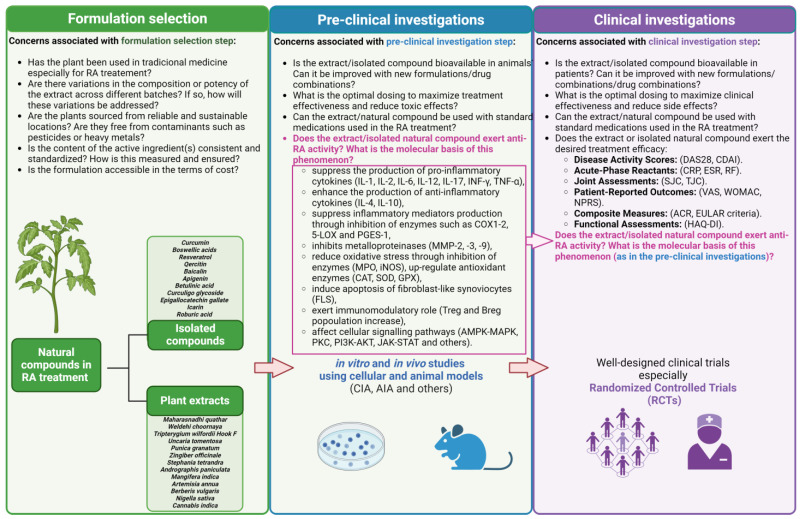
Workflow of plant-derived anti-RA drug discovery endeavors from the selection of the compound/plant extract of interest, through pre-clinical investigations to clinical application.

In addition, the standardization of preparations containing natural compounds poses a significant challenge due to the potential influence of variability in the composition and quality of commercially accessible products on their safety and effectiveness. Ensuring consistency in product quality necessitates the adoption of rigorous quality control protocols and standardized manufacturing procedures. To ascertain the effectiveness, safety, and most suitable dosage protocols of naturally derived compounds, it is imperative to conduct meticulously planned and extensive clinical trials. To evaluate the clinical effectiveness of these compounds in individuals diagnosed with RA, it is crucial to carry out randomized controlled trials that adhere to standardized protocols, incorporate adequate sample sizes, and encompass extended follow-up periods. The necessity of performing comparative analyses between conventional therapies and combination therapy trials is paramount to assessing their potential benefits. It is of utmost importance to undertake a thorough examination of the safety and potential interactions with other pharmaceutical substances, as provided in Figure 7.

## Figures and Tables

**Table 1:** Pre-clinical and clinical studies of natural compounds: curcumin, bosewllic acids, resveratrol, quercitin, and baicalin in the treatment of RA.

Natural Compound	Type of Study (Preclinical/Clinical)	Study Groups/Dosage Regimen	Biological/Clinical Effects	PMID
Curcumin	Clinical (Randomized Pilot Study)	(1) curcumin (BCM-95) 500 mg or (2) curcumin (BCM-95) 500 mg + 50 mg diclofenac sodium (3) 50 mg diclofenac sodium over a period of 8 weeks	The curcumin group exhibited the highest percentage of improvement in overall DAS and ACR (ACR 20, 50, and 70) scores. The administration of curcumin was determined to be safe and did not result in any adverse events.	22407780
	Clinical (Randomized, Double-Blind, Placebo-Controlled, Two-Dose, Three-Arm, and Parallel-Group Study)	(1) placebo, (2) curcumin 250 mg (3) curcumin 500 mg twice daily for 90 days.	Curcumin groups showed significant changes in their clinical symptoms at the end of the study, as evidenced by improvements in ESR, CRP, VAS, RF, DAS28, and ACR responses compared to the placebo group. Curcumin was determined to be safe and did not result in any adverse events.	28850308
	Clinical (Randomized, Double-Blind, Controlled Trial)	(1) curcumin nanomicelle (40 mg) (2) placebo group 3 times a day for 12 weeks	The administration of curcumin nanomicelles resulted in certain favorable alterations in DAS-28 and SJC, albeit without statistical significance.	31482684
	Clinical (Randomized, Double-Blind, Placebo-Controlled Clinical Trial)	(1) curcumin 500 mg or (2) placebo once a day for 8 weeks.	Supplementing with curcumin resulted in a significant reduction in various health markers, including HOMA-IR, ESR, and CRP, as well as weight, BMI, and waist circumference, in the patients when compared to those who received a placebo.	35178811
	Clinical (Randomised Controlled Trial)	(1) curcumin 180 mg/day (2) curcumin 180 mg/day with strengthening exercises 3 sessions/week/24 weeks	Curcumin supplementation with strengthening exercises led to a significantly higher reduction in RF, ESR, CRP, WOMAC pain, and stiffness.	36474395
Boswellic acids	Pre-clinical (in vitro/in vivo)	3-α-o-acetoxy-4β-amino-11-oxo-24-norurs-12-ene (BA-25) In vitro: 10 μM (BA-25) In vivo: (1) MTX (0.3 mg/kg) combination (2) BA-25 + MTX (10 mg/kg + 0.3 mg/kg) from 15th to 34th day.	In vitro: a significant decrease in NO, ROS, TNF-α, and IL-6 production with the combination treatment in comparison to MTX alone in LPS-stimulated RAW-264.7 cells. In vivo: combination therapy restored LPS-induced increases in pro-inflammatory cytokines. There were no notable alterations in the hematological and serum biochemical markers between the combination group and the vehicle group. Furthermore, BA-25 exhibited rapid absorption and a high volume of distribution.	37820446
	Clinical (Double-Blind Pilot Study)	9 tablets of (1) boswellic acid (3600 mg) (2) placebo daily in addition to previous therapy for 12 weeks	There were no subjective, clinical, or laboratory parameters that exhibited a substantial or clinically meaningful change from the initial state, nor were there any differences seen between the two groups at any stage during the study.	9566100
Resveratrol	Pre-clinical (in vitro/in vivo)	Mice with CIA were given resveratrol injections directly into their abdominal cavity every day for 10 days. The injections started either on day 10 to avoid the onset of arthritis, or on day 23 to treat the arthritis after it had already developed following the initial immunization.	Both preventive and curative treatments of resveratrol reduced clinical symptoms and bone degradation in mice with CIA. The protective benefits against arthritis were linked to significantly decreased levels of pro-inflammatory cytokines and collagen-specific IgG in the bloodstream. Additionally, there was a decrease in the number of Th17 cells and the generation of IL-17 in the draining lymph nodes.	21953348
	In vitro	100 μM	Resveratrol was found to induce RA synovial cell (MH7A) apoptosis. This was achieved by activating caspase-9 and caspase-3. Resveratrol also disrupted mitochondrial function, leading to a decrease in the production of BCL-X(L), a protein that promotes cell survival. This disruption allowed cytochrome c to be released from the mitochondria into the cytosol. These effects were dependent on the presence of SIRT1, a protein involved in various cellular processes.	20697895
	Pre-clinical (in vitro/in vivo)	FLSs isolated from RA patients treated with 50–200 μM of resveratrol	The activation of caspase-8 is necessary to initiate resveratrol-induced apoptotic signaling in RA FLS through the participation of the mitochondrial pathway. Exposure to resveratrol resulted in significant apoptotic cell death, characterized by the activation of caspase-9 and -3, cleavage of poly ADP-ribose polymerase (PARP-1), release of mitochondrial cytochrome c, and translocation of apoptosis-inducing factor (AIF) to the nucleus. These events occurred through both caspase-dependent and caspase-independent signaling pathways. It was observed that the activated caspase-8 initiated mitochondrial apoptotic events by causing BID cleavage without affecting the levels of the apoptosis regulator BAX (BAX) or BCL2.	18276737
	Clinical (Randomized Controlled Trial)	(1) resveratrol capsule of 1 g with the conventional treatment (2) control group that received regular treatment for 3 months	The resveratrol-treated group showed a substantial decrease in DAS28. In addition, the levels of specific biochemical markers, including CRP, ESR, undercarboxylated osteocalcin, MMP3, TNF-α, and IL-6, were considerably reduced in patients who had received resveratrol.	29611086
Quercitin	Clinical (Randomized Double-Blind Clinical Trial)	(1) quercetin group (500 mg/day), (2) placebo group (500 mg/day) for 8 weeks.	No significant differences were observed between the quercetin and placebo groups in terms of markers of oxidative stress, such as total antioxidant capacity, oxidized low-density lipo-protein, MDA, and CRP, as well as blood pressure.	24829713
	Clinical (Randomized Double-Blind Clinical Trial)	(1) quercetin group (500 mg/day), (2) placebo group (500 mg/day) for 8 weeks.	The quercitin group had a decrease in early morning stiffness, morning pain, and discomfort following activity. Additionally, there was a reduction in plasma TNF-α levels as well as DAS-28 and HAQ scores.	27710596
	Clinical	glucosamine-chondroitin-quercetin glucoside (GCQG) supplement treatment (consisting of glucosamine hydrochloride (1200 mg/day), shark cartilage powder (300 mg/day) containing 75–111 mg of chondroitin, and quercetin (45 mg/day) for three months	No improvements in pain symptoms, daily activities (e.g., walking), VAS, or changes in the synovial fluid properties concerning the protein concentration, the molecular size of hyaluronic acid, or chondroitin 6-sulphate concentration.	19202302
	Clinical (Randomized, Placebo-Controlled, Double-Blind, Three-treatment Cross-over Design Trial)	Quercetin + vitamin C (166 mg + 133 mg/capsule), alpha-lipoic acid (300 mg/capsule), or placebo for 4 weeks, 3 capsules/day. Each treatment period consisted of 4 weeks with two weeks of wash-out period before the subject started the next supplementation.	There were no notable variations observed in the levels of pro-inflammatory cytokines (TNF-α, IL-1β, IL-6) and CRP in the serum of patients across different therapies. There was no significant difference in illness severity assessments between treatments, while quercetin supplementation showed a tendency to decrease VAS.	19571161
Baicalin	Pre-clinical (in vitro/in vivo)	Murine adjuvant-induced arthritis model: during postimmunization days 14 to 21, mice were injected intraperitoneally with either 100 μL of baicalin solution at a dosage of 100 mg/kg or an equal volume of phosphate buffered saline PBS as a control. In vitro: 20 μM	Baicalin suppressed the increase in splenic Th17 cell population in vivo and blocked the adherence of lymphocytes mediated by IL-17 to cultured synoviocytes. Baicalin suppressed the expression of ICAM-1, VCAM-1, IL-6, and TNF-α mRNA in cultured synoviocytes. Baicalin reduces joint inflammation induced by IL-17, which is likely produced by an increased number of splenic Th17 cells in experimental arthritis.	23840239
	Pre-clinical (in vivo)	CIA rats were treated with baicalin (50, 100, or 200 mg/kg) once daily for 30 days.	Administration of baicalin through intraperitoneal injection for 30 days effectively inhibited the observable symptoms of CIA, including reduced functionality and inflammation in the paws. Furthermore, it reduced collagen-induced joint inflammatory damage and suppressed the release of TNF-α and IL-1β in both rat synovium and synoviocytes derived from RA patients. Additional mechanistic studies demonstrated that baicalin inhibits the production and phosphorylation of the NF-κB p65 protein in synovial tissue and synoviocytes isolated from RA patients. In addition, baicalin reduced the acetylation of NF-κB p65, which is inversely related to the increase in SIRT1 expression caused by baicalin under the same conditions.	24893986
	Pre-clinical (in vivo)	(1) control group, (2) RA model + saline treatment group (3) RA model + baicalin treatment group (30 mg/kg baicalin for five consecutive days)	RA was generated in mice by intraperitoneally injecting them with a collagen II monoclonal antibody cocktail at a concentration of 5 mg/kg dissolved in PBS for 10 consecutive days. The mice were administered LPS intraperitoneally at a dosage of 100 μg per mouse in 200 μL sterile PBS either on day 1 or day 4. Baicalin exerted a notable reduction in disease activity in a mouse model, as evidenced by the decrease in pressure pain thresholds and clinical arthritis scores. The expression of TNF-α, IL-1β, IL-6, MMP-2, MMP-9, and inducible enzymes (iNOS, COX-2) was generally suppressed. Furthermore, the administration of baicalin resulted in the apoptosis of synovial fluid monocytes and significantly decreased the expression of JAK1/STAT3, but not MAPKs, in the synovium.	30562763
	Pre-clinical (in vivo)	(1) normal group (normal saline), (2) model group (normal saline), (3) dexamethasone group (0.125 mg/kg dexamethasone), (4) low-dose baicalin group (50 mg/kg baicalin), (5) medium-dose baicalin group (100 mg/kg baicalin) and 6) high-dose baicalin group (200 mg/kg baicalin).	Baicalin suppressed the expression of TNF-a, IL-6, IL-1, IL-17, COX2, and COX1 in the synovial tissue. Varying concentrations of baicalin decreased the levels of autophagy-related proteins while increasing the levels of BAX proteins in the synovial tissue of adjuvant arthritis model rats.	35124596
	Clinical (Randomized, Double-blind, Placebo-Controlled Trial)	500 mg baicalin or placebo orally every day for 12 weeks	Baicalin demonstrated efficacy in lowering blood lipids and reducing inflammation in patients diagnosed with both coronary artery disease and RA, as evidenced by CRP and EULAR improvements.	29935544

**Table 2:** Clinical studies of plant extracts in RA treatment.

Herb/Compound	Duration	Dosage/Form	Outcome Measures	Results	PMID
Maharasnadhi Quathar (MRQ)	3 months	-	SOD, GPX, CAT, TBARS, serum iron, hemoglobin, TIBC	Increased SOD by 44.6%, GPX by 39.8%, CAT by 25.2%; Reduced TBARS by 34%; Improved serum iron by 26.8%, hemoglobin by 24.8%, TIBC by 16.1%	11448551
Weldehi Choornaya (WC)	3 months	-	SOD, GPX, CAT, TBARS, serum iron, hemoglobin, TIBC	No notable enhancement in SOD, GPX, or CAT; Reduced TBARS by 21.8%	11448551
*Tripterygium* *wilfordii* Hook F (TWHF)	-	Varied doses	Safety, ACR20/70, DAS28, EULAR	High dose (360 mg/day) showed efficacy; 570 mg/day was well-tolerated; TWHF extract, administered at a dose of 60 mg three times daily, resulted in a significantly higher rate of achieving the ACR20 (20% improvement) compared to sulfasalazine over 24 weeks; TWHF monotherapy (20 mg three times a day) was comparable to that of MTX monotherapy (12.5 mg once a week) in managing disease activity in patients with active RA. Additionally, the combination of MTX and TWHF was found to be superior to MTX monotherapy for 12 or 24 weeks.	11669150 12124856 19687490 24733191 29636089
*Uncaria tomentosa* (UT)	52 weeks	-	Sore joints, swollen joints, safety	Decrease in sore joints; No major adverse effects	11950006
*Punica granatum* (Pomegranate)	8 weeks	250 mg twice daily	DAS28, HAQ, CRP, MMP3, MDA, GPX, ESR	Decreased DAS28; improved blood biomarkers of inflammation, and oxidative stress	27577177
*Zingiber officinale* (Ginger)	8 weeks	500 mg twice daily	Total anti-oxidant capacity, MDA, HAQ	Improved oxidative stress markers and HAQ	32159257
*Stephania* *tetrandra*	12 weeks	-	Granulocyte count, lipid peroxide, elastase	Reduced granulocyte count, lipid peroxide, and elastase activities; improved inflammation biomarkers	15103675
Cannabis (Sativex)	5 weeks	Oromucosal spray	Pain, morning stiffness, sleep quality, DAS28	Reduced pain and disease activity	16282192
*Andrographis paniculata* (Andrographis)	14 weeks	30% andrographolides extract	Pain, joint swelling, tenderness, ACR, EULAR, SF36	Reduction in joint pain, swelling, and tenderness; not statistically significant	19408036
*Mangifera indica* (Vimang)	180 days	900 mg daily	DAS28, VAS, HAQ, ESR	Significant improvement in DAS28; reduced NSAID use	24344049
*Artemisia annua*	12 weeks	150 mg twice daily	Pain, stiffness, functional limitations	Reduced pain over 12 weeks	26631103
*Berberis vulgaris* (Barberry)	12 weeks	1000 mg/twice daily	IL-2, IL-4, IL-10, IL-17	Decreased IL-17, increased IL-10; reduced RA intensity	32914483
*Nigella sativa* (Black Seed)	2 months	500 mg/twice daily	DAS28, hs-CRP, CD4+, CD8+, IL-10, TNF-α, MDA, NO	Decreased DAS28, CRP, increased CD4(+)/CD8(+) ratio	27100726
Willow Bark (*Salix* spp.)	-	Standardized extract	WOMAC pain score, VAS	No positive effects	15517622

**Table 3:** Pre-clinical studies of natural compounds in the RA treatment.

Compound	Class	Mechanisms of Action	PMID	Pharmacokinetics/Toxicity	PMID
Apigenin	Flavonoid	Inhibits ERK1/2 pathway. Reduces production of IL-1β, IL-6, TNF-α. Inhibits the COX2 enzyme. Inhibits collagenase activity. Decreases NO production. Reduces expression of COX-2, VCAM-1, ICAM-1, E-selectin. Inhibits angiogenesis and osteoclastogenesis via the VEGF/VEGFR and RANKL/osteoprotegerin pathways	24685587 31132731 19647729 22189539 35660352 21535545 18038911	Currently, there is limited evidence indicating any harmful effects caused by apigenin. The pharmacokinetic behavior of apigenin influences its distribution in tissues and its bioactivity. The O-glycosylation or C-glycosylation of apigenin can have varying effects on its metabolism, which in turn might impact its antioxidant capability and biological advantages. The reason for this is likely because apigenin, despite its many beneficial benefits, has a very low solubility in water (1.35 μg/mL) and high permeability. Nevertheless, apigenin is widely regarded as safe, even when taken in large amounts, and there have been no reports of any harmful effects.	30875872
Betulinic Acid (BA)	Triterpenoid	Suppresses growth, motion, and infiltration of FLSs. Reduces TNF-α exposure effects, MMP expression, and inflammatory cytokines (IL-6, IL-8) production. Inhibits the AKT/NF-κB pathway. Inhibits migration, invasion, and actin cytoskeleton rearrangement in FLSs. Reduces mRNA levels of IL-1β, IL-6, IL-8, and IL-17A. Reduces NF-κB signal pathway activation. Lowers IL-1β, IL-6, and TNF-α levels. Suppresses VEGF and TGF-β transcription. Reduces NF-κB pathway protein expression.	30367550 30553912 32718263	Research has indicated that BA does not have any notable adverse effects. At a dosage of 50 μg/mL, BA did not impede the growth of normal peripheral blood cells. The limited water solubility of BA impedes its potential use as a medication. Modifications at carbon positions C-3, C-20, and C-28 of the molecule have the potential to enhance solubility while maintaining pharmacological action.	34388528
Curculigo glycoside	Steroidal saponin	Reduces inflammatory markers (TNF-α, IL-1β, IL-6, IL-12, IL-17A). Modulates the JAK/STAT/NF-κB signaling pathway. Reduces EGFR, MEK1, MMP2, FGFR1, and MCL1 expression. Inhibits inflammatory cell infiltration, pannus formation, and cartilage damage. Suppresses MMP9, JUN, and PTGS2 expression.	26637957 30664158 33273808 37957674	Some studies have shown that curculigo glycoside use in living organisms can be challenging, mainly due to low oral bioavailability. Curculigo glycoside passes through some metabolic changes, most of which are related to phase I and phase II biotransformation events. As a phenolic glycoside, curculigo glycoside can undergo phase I metabolic processes involving oxidation, hydrolysis, or reduction. This leads to the breakdown of ester and phenolic glycoside bonds. Furthermore, oxidation or reduction events may be initiated by phenolic hydroxyl groups and their derivatives, hydroxymethyl and benzoyloxy groups. Curculigoside demonstrated fast distribution, wide tissue uptake, and limited absorption into systemic circulation in rats. The absolute bioavailability values for oral dosages of 100, 200, and 400mg/kg were determined to be 0.38%, 0.22%, and 0.27%, respectively. According to the tissue distribution study, curculigoside was found in many organs, including the heart, lung, spleen, intestine, stomach, kidney, thymus, liver, brain, testis, and bone marrow, after being orally given at a dose of 150 mg/kg. Its broad distribution suggests that it can pharmacologically affect target tissues following pharmacokinetic optimization. Curculigoside specifically decreased the activity of CYP1A2, CYP2C8, and CYP3A4, but not that of other CYP isoforms. While extensive toxicological studies of curculigoside were not conducted, acute toxicity studies revealed that water extracts of *Curculigo orchioides* did not affect animal mortality, even at a dosage that was 1384 times higher than the recommended clinical dose (3–9 g daily). The LD_50_ of ethanol plant extracts was 215.9 g/kg, which was 1439 times higher than the recommended dose. Similarly, long-term toxicity experiments revealed that administering the ethanol extract of *Curculigo orchioides* to rats at a dose of 120 g/kg for 6 months resulted in damage to the liver, kidney, and reproductive organs. Administration of either 30 g/kg or 60 g/kg doses over a long time did not result in any toxicological consequences.	27819189 23562803 34205154 25549926
Epigallocatechin Gallate (EGCG)	Flavonoid (Catechin)	Reduces IL-1β, TNF-α, and IL-6 production. Neutralizes ROS. Modulates anti-oxidant processes and immune cell activity. Inhibits MMPs, cathepsins, and CCL2 production. Stimulates indoleamine-2,3-dioxygenase-producing dendritic cells and T regs. Activates ERK, NRF2, and HO-1. Suppresses STAT3 activation. Enhances PI3K and AKT activity. Reduces MCL-1 expression.	18496696 18796608 23871988 28532672 31746064 26379475 16869002 22186621 18576345 18821707 26957211 19404960	EGCG experiences challenges in its therapeutic application due to its limited oral bioavailability, which is attributed to its low absorption in the intestines and its short retention period or lack of stability. EGCG has been studied in doses ranging from 150 to 400 mg per day, with oral administration being the most commonly used method in animal models and clinical trials. On the other hand, it has been suggested that daily doses ranging from 140 mg to 1000 mg can cause damage to the liver. Lambert et al. examined the hepatotoxic effects of high dosages of EGCG in male CF-1 mice. An administration of a single dose of EGCG at a dosage of 1500 mg/kg, given orally, resulted in a 138-fold increase in plasma ALT levels and caused a decrease in mouse lifespan by 85%. Administration of EGCG once a day resulted in a hepatotoxic response. After administering two daily doses of 750 mg/kg EGCG, the levels of ALT in the plasma increased by 184 times. After receiving EGCG treatment, hepatic necrosis was observed. This was accompanied by increased levels of hepatic lipid peroxidation (5-fold change) and liver damage. The safe intake limit of 338 mg of EGCG/day for adults was determined based on the analysis of toxicological and human safety data for tea preparations ingested as a solid bolus dosage. According to evidence on adverse effects in humans, a recommended safe dose was estimated as 704 mg of EGCG per day (for tea preparations in a drinkable form).	29580974 16387402 19883714 32140423 9311619 22016611 32438698 29799486 37446908
Icarin and icaritin (metabolite)	Flavonoid glycoside	Reduces β3 integrin, cathepsin K, and RANKL expression. Suppresses the MAPK and NF-κB pathways. Alleviates bone degradation, suppresses proliferation, and promotes apoptosis of FLS cells. Reduces TNF-α, IL-1β, and IL-6 secretion.	35676785 23746958 27199510 26816641 25374443 37647355 33155655 34678240	Recent clinical trials suggest that icaritin has a reasonably good level of safety, however, it may lead to modest side effects such as skin rash and diarrhea. Icaritin suppresses the activity of UGT1A1, 1A3, 1A4, 1A7, 1A8, 1A10, 2B7, and 2B15 enzymes. This suggests that this chemical has the potential to interact with other medications that are processed by UGT enzymes.	30922248 30522143 34873891 38886878
Roburic Acid (RBA)	Tetracyclic triterpenoid	Inhibits mPGES-1 and COX1/2 enzymes. Suppresses the MAPK and NF-κB pathways. Direct association with TNF-α, prevents its binding to TNF-R1. Suppresses osteoclast development. Inhibits TRAF6 and NF-κB activity. Increases HO-1 and ARE activity.	24844534 20188172 28761990 35222445 34796915 37500654	No data.	

## Abbreviations

ACPA	anti-citrullinated peptide/protein antibodies
ACR	American College of Rheumatology
ADA	anti-drug antibodies
AIA	adjuvant-induced arthritis
AIF	apoptosis-inducing factor
AKBA	acetyl keto boswellic acid
AKT	AKT serine/threonine kinase
AMPK	5′-AMP-activated protein kinase catalytic subunit alpha-1
AP-1	adaptor protein 1
APC	antigen-presenting cell
ARE	antioxidative response element
BAX	apoptosis regulator BAX
BCL-2	apoptosis regulator Bcl-2
Breg	B regulatory cells
CA	curculigoside A
CAT	catalase
CBD	cannabidiol
CCL-1	monocyte chemoattractant protein-1
CCR6	chemokine receptor 6
CDAI	Clinical Disease Activity Index
CIA	collagen-induced arthritis
COX	cyclooxygenase
CRP	C-reactive protein
CTLA4	cytotoxic T-lymphocyte-associated protein 4
CYP	cytochrome P450
DAS	Disease Activity Score
DMARDs	disease-modifying antirheumatic drugs
EGCG	epigallocatechin gallate
EGFR	epidermal growth factor receptor
ERK	extracellular signal-regulated kinase
ESR	erythrocyte sedimentation rate
EULAR	European League Against Rheumatism
FGFR	fibroblast growth factor receptor 1
FLS	fibroblast-like synoviocytes
GCQG	glucosamine-chondroitin-quercetin glucoside
GPX	glutathione peroxidase
GSH	glutathione
GWAS	genome-wide association studies
HAQ-DI	Health Assessment Questionnaire Disability Index
HLA	human leukocyte antigen
HO-1	heme oxygenase-1
HOMA-IR	Homeostatic Model Assessment of Insulin Resistance
ICAM-1	intercellular adhesion molecule 1
IL	interleukin
IL-23R	interleukin-23 receptor
INF-γ	interferon-γ
iNOS	inducible nitric oxide synthase
IRF5	interferon regulatory factor 5
IκBα	NF-kappa-B inhibitor alpha
JAK	Janus kinase
LPO	lipid peroxidase
LPS	lipopolysaccharide
MAPK	mitogen-activated protein kinase
MCL-1	myeloid cell leukemia 1
MDA	malondialdehyde
M-EC	membrane-camouflaged nanoparticles
MEK1	dual specificity mitogen-activated protein kinase kinase 1
MMP	matrix metalloproteinase
mPGES-1	microsomal prostaglandin E2 synthase-1
MPO	myeloperoxidase
MRQ	Maharasnadhi quathar
MTX	methotrexate
NFATc1	nuclear factor of activated T cells 1
NF-κB	nuclear factor kappa B
NO	nitric oxide
NOEL	no observed effect level
NPRS	Numeric Pain Rating Scale
NRF-2	nuclear factor erythroid 2-related factor 2
NSAIDs	nonsteroidal anti-inflammatory drugs
OA	osteoarthritis
PADI4	protein-arginine deiminase type-4
PARP-1	poly ADPribose polymerase
PBS	phosphate buffered saline
PGE-2	prostaglandin E2
PI3K	phosphatidylinositol 4,5-bisphosphate 3-kinase
PKC	protein kinase C
PROs	patient-reported outcomes
PTPN22	phosphatase non-receptor type 22
RA	rheumatoid arthritis
RANKL	receptor activator for nuclear factor κB ligand
RBA	roburic acid
RF	rheumatoid factor
RNS	reactive nitrogen species
ROS	reactive oxygen species
SE	shared epitope
SIRT1	sirtuin 1
SJC	swollen joint count
SOD	superoxide dismutase
STAT	signal transducer and activator of transcription
TBARS	thiobarbituric acid reacting substances
TGF-β	transforming growth factor β
THC	tetrahydrocannabinol
TJC	tender joint count
TNF-R1	tumor necrosis factor receptor 1
TNF-α	tumor necrosis factor-alpha
TRAF1/6	TNF receptor-associated factor 1/6
Tregs	regulatory T cells
UT	Uncaria tomentosa
VAS	Visual Analog Scale
VCAM-1	vascular cell adhesion protein 1
VEGF	vascular endothelial growth factor
VEGFR	vascular endothelial growth factor receptor
WC	Weldehi choornaya
WOMAC	Western Ontario and McMaster Universities Osteoarthritis Index
